# Laterality of Poststroke Cortical Motor Activity during Action Observation Is Related to Hemispheric Dominance

**DOI:** 10.1155/2018/3524960

**Published:** 2018-05-28

**Authors:** Sook-Lei Liew, Kathleen A. Garrison, Kaori L. Ito, Panthea Heydari, Mona Sobhani, Julie Werner, Hanna Damasio, Carolee J. Winstein, Lisa Aziz-Zadeh

**Affiliations:** ^1^University of Southern California, Los Angeles, CA, USA; ^2^Yale University, New Haven, CT, USA; ^3^California State University, Dominguez Hills, Carson, CA, USA; ^4^Children's Hospital Los Angeles, Los Angeles, CA, USA

## Abstract

**Background:**

Increased activity in the lesioned hemisphere has been related to improved poststroke motor recovery. However, the role of the dominant hemisphere—and its relationship to activity in the lesioned hemisphere—has not been widely explored.

**Objective:**

Here, we examined whether the dominant hemisphere drives the lateralization of brain activity after stroke and whether this changes based on if the lesioned hemisphere is the dominant hemisphere or not.

**Methods:**

We used fMRI to compare cortical motor activity in the action observation network (AON), motor-related regions that are active both during the observation and execution of an action, in 36 left hemisphere dominant individuals. Twelve individuals had nondominant, right hemisphere stroke, twelve had dominant, left-hemisphere stroke, and twelve were healthy age-matched controls. We previously found that individuals with left dominant stroke show greater ipsilesional activity during action observation. Here, we examined if individuals with nondominant, right hemisphere stroke also showed greater lateralized activity in the ipsilesional, right hemisphere or in the dominant, left hemisphere and compared these results with those of individuals with dominant, left hemisphere stroke.

**Results:**

We found that individuals with right hemisphere stroke showed greater activity in the dominant, left hemisphere, rather than the ipsilesional, right hemisphere. This left-lateralized pattern matched that of individuals with left, dominant hemisphere stroke, and both stroke groups differed from the age-matched control group.

**Conclusions:**

These findings suggest that action observation is lateralized to the dominant, rather than ipsilesional, hemisphere, which may reflect an interaction between the lesioned hemisphere and the dominant hemisphere in driving lateralization of brain activity after stroke. Hemispheric dominance and laterality should be carefully considered when characterizing poststroke neural activity.

## 1. Introduction

Despite intensive research and clinical efforts, stroke remains a leading cause of physical disability worldwide [1], and there is an urgent need for improved poststroke rehabilitation strategies. Many studies have suggested that increased levels of activity in the ipsilesional hemisphere after stroke are associated with enhanced recovery [2–4]. Functional magnetic resonance imaging (fMRI) studies in individuals with stroke suggest that greater blood oxygen level-dependent (BOLD) activity in the contralateral (ipsilesional) hemisphere during a task with the impaired upper limb—a pattern consistent with typical motor control—is associated with better motor outcomes [4–6]. Poststroke therapeutic techniques have therefore aimed at promoting motor recovery by increasing activity in the ipsilesional hemisphere and decreasing activity in the contralesional hemisphere [7–10]. However, poststroke motor outcomes using such approaches remain variable, suggesting that other factors influence recovery beyond the level of brain activity in the ipsilesional hemisphere.

One factor that has been largely overlooked in stroke studies is the role of motor dominance relative to the lesioned hemisphere. Studies often discuss findings related to the ipsilesional or contralesional hemisphere, without distinguishing whether the ipsilesional hemisphere is the dominant or nondominant hemisphere prior to stroke. However, whether stroke occurs in the dominant or nondominant hemisphere can impact recovery in multiple ways, including impacting the pattern of brain activity achieved in poststroke therapy [11–13]. Research has shown clear hemispheric differences in the specialization of motor control, with differences in the performance of motor actions after stroke related to the lesioned hemisphere. Winstein and Pohl (1995) reported that individuals with left hemisphere stroke showed deficits in open-loop, motor planning aspects of movement, whereas individuals with right hemisphere stroke showed deficits in closed-loop, feedback-based aspects of movement [14]. Another study showed that in an arm-reaching task, individuals with left hemisphere stroke had difficulty controlling the direction of movement, whereas individuals with right hemisphere stroke had a tendency to overshoot their targets [11]. These studies and others suggest that there is hemispheric specialization in a distributed motor control scheme, where the left hemisphere is responsible for optimizing and predicting dynamic aspects of movement, and the right hemisphere is responsible for movement accuracy and stability [15]. There are thus likely differences in task-related brain activity depending on the dominance of the lesioned hemisphere. Better understanding the relationship between motor dominance, hemisphere of stroke, and brain activity is critical because it could enable greater personalization of interventions in stroke neurorehabilitation and allow us to better understand the neural mechanisms underlying deficits following stroke.

Here, we hypothesized that the motor dominant hemisphere might in fact drive poststroke brain activity during action observation more strongly than the side of the stroke lesion. We specifically evaluated brain activity in the action observation network (AON), as it is a brain network typically engaged through both the observation and performance of actions and is comprised of cortical motor regions in the premotor and parietal cortices [16]. Importantly, activity in the AON can be elicited simply through action observation, so even individuals with moderate to severe upper arm paresis can complete the task. Action observation therapy (AOT), in which individuals with stroke observe another person performing actions (e.g., through videos) before or during actual physical practice of those actions, has been proposed as a way to enhance the effects of occupational or physical therapy [17–20]. Behavioral studies examining outcomes of AOT with occupational or physical therapy show modest improvements in poststroke motor recovery when compared to traditional therapy alone [17–19, 21, 22]. Researchers hypothesize that action observation may enhance plasticity in the same motor pathways responsible for action execution [23]. The AON has also been shown to be active during action observation in individuals after stroke [24]. In particular, activity in the AON was found to be lateralized to the ipsilesional, dominant hemisphere in individuals with motor dominant, left hemisphere stroke who observed actions being performed by the counterpart to their own paretic right arm [24]. However, since the left hemisphere was both the ipsilesional and motor dominant hemisphere, it was not possible to distinguish whether action observation drives activity in the ipsilesional hemisphere or in the motor dominant hemisphere.

The present study was designed with a primary aim of improving our understanding of the effects of motor dominance versus side of lesion on AON activity in individuals after stroke. We recruited individuals who were left hemisphere dominant (right handed) prior to stroke and had nondominant, right hemisphere stroke. Using the same fMRI protocol as in the earlier AON stroke study [24], we tested whether individuals with nondominant right hemisphere stroke had greater AON activity during action observation in the ipsilesional (right) hemisphere or in the dominant (left) hemisphere. We compared these data to the dominant left hemisphere stroke group and an age-matched control group from the earlier study [24]. We predicted that if action observation drives activity in the ipsilesional hemisphere, the right hemisphere stroke group should show greater activity in the right hemisphere, whereas if action observation drives activity in the motor dominant hemisphere, the right hemisphere stroke group should show greater activity in the left hemisphere.

## 2. Methods

### 2.1. Subjects

The current analysis included 36 individuals who were right-handed (left hemisphere motor dominant) as determined by the Edinburgh Handedness Inventory [25]. There were 24 participants with chronic stroke and moderate-to-severe upper extremity motor impairments and 12 nondisabled, age-matched controls. Both the nondisabled controls and 12 individuals with dominant left hemisphere stroke were included in an earlier study [24]. In the current study, 12 additional individuals with nondominant right hemisphere stroke were recruited from community centers. All participants gave informed consent in accordance with institutional guidelines approved by the University of Southern California Institutional Review Board. All individuals were right handed (prior to stroke), had normal or corrected-to-normal vision, and were safe for MRI. Additional inclusion criteria for individuals with stroke was chronic (>3 months since stroke onset), middle cerebral artery stroke, with no prior history of stroke, moderate-to-severe upper extremity impairment as determined by a phone screening form in which participants indicated difficulty moving their hand or arm for functional tasks, and no apraxia. For all participants, mean age (including nondisabled controls) was 63 ± 13 years and did not differ between groups (*F*(2, 33) = 0.94, *p* = 0.40). For participants with stroke, average time since stroke was 80 ± 58 months and did not differ between right and left hemisphere stroke groups (*t*(22) = −0.17, *p* = 0.61). Stroke characteristics are described in Table 1.

### 2.2. fMRI Data Acquisition

All scanning was completed on the same 3T Siemens Trio MRI scanner at the University of Southern California Dornsife Neuroimaging Center, using the scan parameters and task as described in Garrison et al. [24]. Functional images were acquired with a T2^∗^-weighted gradient echo sequence (repetition time [TR]/echo time [TE] = 2000/30 ms, 37 slices, voxel size 3.5 mm isotropic voxels, and flip angle 90°); anatomical images were acquired with a T1-weighted magnetization-prepared rapid gradient-echo (MPRAGE) sequence (TR/TE = 2350/3.09 ms, 208 1 mm slices, 256 × 256 mm, and flip angle 10°).

The fMRI paradigm was a block design in which participants either observed either videos of right hand actions, videos of left hand actions, and images of a still hand (control condition) or rested. For the action observation conditions, videos depicted a mean-age-matched nondisabled control actor grasp objects with either their right hand or their left hand, as previously described [24]. Actions were adapted from the Wolf Motor Function Test (WMFT, Wolf et al. [26]) and included (1) pick up pencil, (2) pick up paperclip, (3) stack checkers, and (4) flip cards (see Supplementary Figure S1 for an example). Each video was 3 s long, and each block consisted of four videos shown in a randomized order for a total block length of 12 s. The control condition (observation of a still hand) was also presented in 12 s blocks with 4 still images of either a left or a right hand shown for 3 s each in a randomized order. All blocks were repeated 15 times and randomized across three 6-minute runs.

Participants were instructed to remain still and watch the actions of the actor as they would be asked to imitate each action after the scanning session. To ensure attention, participants were asked questions about the videos at the end of each run (e.g., “In the last video you saw, which hand did the actor use?”).

### 2.3. fMRI Analysis

#### 2.3.1. Preprocessing and Analyses

Functional neuroimaging data analysis was carried out using FEAT (FMRI Expert Analysis Tool) Version 6, part of FSL (FMRIB's Software Library, http://www.fmrib.ox.ac.uk/fsl). Registration to high-resolution structural and standard space images was carried out using FLIRT [27, 28]. The following preprocessing steps were applied: semimanual skull stripping of the anatomical image using BET [29], motion correction using MCFLIRT [27], automated nonbrain removal of the fMRI data using BET [29], spatial smoothing using a Gaussian kernel of FWHM 5 mm, grand-mean intensity normalization of the entire 4D dataset by a single multiplicative factor, and high-pass temporal filtering (Gaussian-weighted least-squares straight line fitting, with sigma = 50 s). For each subject, a time-series statistical analysis was carried out using FILM GLM with local autocorrelation correction [30]. These *Z* (Gaussianized T/F) statistic images were then thresholded using clusters determined by *Z* > 3.1 and a (corrected) cluster significance threshold of *p* < 0.05 [31]. A second-level analysis for each subject was conducted, averaged across the three runs, and carried out using a fixed-effects model by forcing the random-effects variance to zero in FLAME (FMRIB's Local Analysis of Mixed Effects) [32–34]. At the group level, analyses were completed using a mixed-effects model that included both fixed effects and random effects from cross session/subject variance in FLAME. Again, *Z* (Gaussianized T/F) statistic images were then thresholded using clusters determined by *Z* > 3.1 and a (corrected) cluster significance threshold of *p* < 0.05. Whole brain analyses examined main effects of right hand action observation, main effects of left hand action observation, and contrasts of right hand action observation versus left hand action observation and left hand action observation versus right hand observation.

An additional aim of the current study was to directly compare new data from the right hemisphere stroke group to the data from the left hemisphere stroke group and nondisabled control group from the earlier study [24]. In order to do this, we reanalyzed all of the earlier data using the preprocessing steps described above to ensure that the same, up-to-date analysis techniques were used in all cohorts.

#### 2.3.2. Region of Interest Analyses

A priori regions of interest (ROIs) included regions of the human AON: inferior frontal gyrus pars opercularis (IFGop) and pars triangularis (IFGtri), the supramarginal gyrus (SMG), and the precentral gyrus (PC) [35]. ROIs were defined anatomically using the probabilistic Harvard-Oxford Atlas included in FSL, with a probability threshold of greater than 25% applied for each ROI. The percent signal change (% SC) within each ROI was extracted for each task condition and each participant using Featquery in FSL.

#### 2.3.3. Laterality Index

A laterality index (LI) was calculated to measure lateralization of brain activity during observation of each hand (right, left) for each group (nondisabled control, right hemisphere stroke, and left hemisphere stroke). LI was calculated as the proportion of active voxels in the left versus right ROI averaged across multiple thresholds [36, 37]. We calculated LI using the proportion of active voxels, rather than percent signal change, based on previous work suggesting that this approach is more robust for lesioned brains (Jansen et al. [37]). The cluster tool in FSL was used to set the different threshold values (*Z* = 1.0, 1.5, 2.3); Fslstats was used to determine the number of active voxels. LI was calculated using the classic formula

(1) $$\begin{array}{c} LI=\frac{\left(left-right\right)}{\left(left+right\right)} \end{array}$$

at each *Z*-threshold for each ROI [37], where LI is equal to left hemisphere activity minus right hemisphere activity divided by left hemisphere activity plus right hemisphere activity. The average of the three LIs at different *Z*-values was then calculated. LI scores range from +1 (all left hemisphere activation only) to −1 (all right hemisphere activation only) and were categorized as either bilateral (∣LI∣ ≤ 0.1), hemisphere dominant (0.1 < ∣LI∣ < 0.2), or hemisphere lateralized (∣LI∣ ≥ 0.2) [37]. Following previous work, the LI and standard error of the mean (SEM) are reported [24]. The complete LI values for each group at each threshold/ROI can be found in Supplementary Table 5 and for each individual at each threshold/ROI in Supplementary Tables 6–9.

#### 2.3.4. ROI-Based Task by Hemisphere by Group Interactions

A three-way ANOVA was carried out in SPSS 22 (IBM Corp., Armonk, NY, USA) to determine the effects of hand observed (right, left), hemisphere of activity (right, left), and group (nondisabled control, right hemisphere stroke, and left hemisphere stroke) for each of our four regions of interest. We applied a Bonferroni correction for multiple comparisons. We also report any significant two-way interactions within the ANOVAs and subsequently tested for simple main effects where appropriate.

#### 2.3.5. Lesion Analyses

Lesions were manually drawn by a trained research assistant following a detailed lesion tracing protocol [38, 39] using MRIcron [40]. Lesion masks were then smoothed using a 2 mm Gaussian kernel. For each subject, a small mask was manually created in the healthy white matter tissue of the contralesional hemisphere. The white matter mask was used to determine the mean and standard deviation of healthy white matter voxel intensities within each subject's anatomical image using fslstats. Each subject's anatomical image was then thresholded at one standard deviation away from the mean white matter intensity, such that voxels with a signal intensity within or above the normal range would be excluded from the final lesion mask. Finally, the volume of the lesion was calculated using fslstats. An independent-sample *t*-test was conducted to compare lesion size between the right hemisphere stroke group and the left hemisphere stroke group. For each ROI, Pearson product-moment correlations were tested between lesion size and LI for that ROI, for each stroke group.

#### 2.3.6. Percent of Lesion Overlap with ROIs

To examine the percent of lesion overlap with each ROI, each individual's binarized lesion mask was normalized to standard space and masked with each ROI using fslmaths. The number of voxels in the overlapping area was obtained using fslstats. The number of voxels in the overlapping area was then divided by the total number of voxels within the ROI to calculate the percent of overlap between the lesion and the ROI for each subject.

### 2.4. Behavioral Assessments

Immediately after the scanning session, participants completed a series of behavioral assessments. Due to the small sample size, behavioral correlations with fMRI data were used as secondary, exploratory analyses. Participants were administered the Wolf Motor Function Test (WMFT) to test the function of the upper extremity in the motor domain [26]. Performance on the WMFT was videotaped and scored by a trained, blinded research assistant for a Functional Ability Scale (FAS) score, ranging from 0 to 5, where 0 = does not attempt movement and 5 = movement is normal. Individuals were also assessed with the Fugl-Meyer Assessment, Upper Extremity (FMA-UE) [41], a measure of poststroke motor impairment. Behavioral assessments were performed by graduate research assistants who were trained in the administration of both the WMFT and FMA-UE assessments.

For each ROI, Spearman's rho correlations were tested between ROI activity and motor scores for the WMFT and FMA-UE (both categorical variables) in SPSS 22. We note that these results are exploratory and report results, noting that correcting for multiple comparisons across ROIs results in a corrected *p* value of *p* = 0.00625 (*p* = 0.05 divided by 8 comparisons).

## 3. Results

In the current study, we aimed to understand whether individuals with nondominant right hemisphere stroke showed greater ipsilesional right hemisphere activity or greater motor dominant left hemisphere activity during action observation. In order to better generalize our findings, we also compared the nondominant right hemisphere stroke group with a dominant left hemisphere stroke group and a nondisabled control group from our earlier study [24].

### 3.1. Between-Group Behavioral Comparisons

Individuals with right hemisphere stroke had significantly lower Fugl-Meyer scores than individuals with left hemisphere stroke had (*t*(22) = −0.67, *p* = 0.02), indicating greater poststroke motor impairments of the upper extremity in the right hemisphere stroke group. Fugl-Meyer scores are reported in Table 1. Similarly, on the WMFT, individuals with right hemisphere stroke showed a trend towards lower FAS scores (*t*(21) = 1.94, *p* = 0.06) than did individuals with left hemisphere stroke, again indicating poorer motor performance. WMFT scores are reported in Table 1, along with all participant demographics.

### 3.2. Whole-Brain fMRI Analyses

Notably, overall, patterns of brain activity during right and left hand action observation were similar between right and left hemisphere stroke groups, despite the groups having motor impairments in opposite hands. Contrasts of right versus left hand action observation, and vice versa, showed similar patterns in the stroke groups and a different pattern in the nondisabled group.

#### 3.2.1. Right Hemisphere Stroke Group

**Figure 1:**
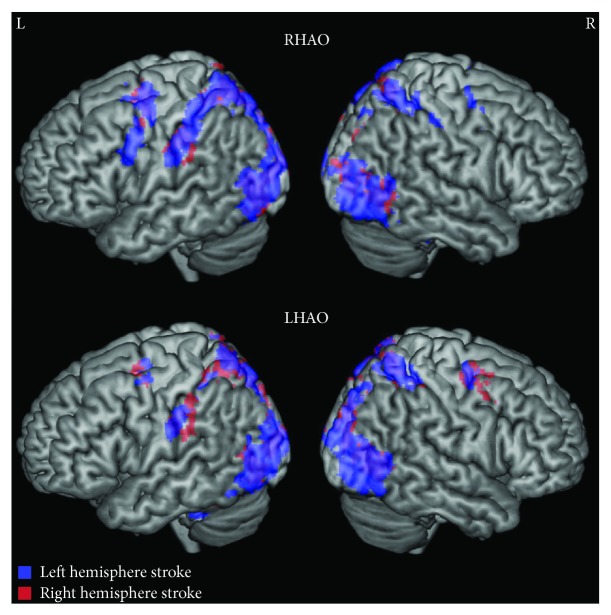
Whole brain activity during right and left action observation for individuals with stroke. While both stroke groups show bilateral activity during right and left hand action observation, activity in the left hemisphere was more extensive regardless of hemisphere of lesion. Top: right hand action observation (RHAO), bottom: left hand action observation (LHAO). Participants with left hemisphere stroke are represented in blue; participants with right hemisphere stroke are represented in red. Overlap between stroke groups is represented in purple. Thresholded at *Z* > 3.1, corrected for multiple comparisons at *p* < 0.05.

For the right hemisphere stroke group, during *right (corresponding to nonparetic) hand action observation*, activity was found in the left premotor cortex, bilateral precentral gyri, bilateral superior parietal lobules, and bilateral occipital cortices, among other areas (Figure 1; Supplementary Table 1). During *left (corresponding to paretic) hand action observation*, activity was again found in the left supramarginal gyrus, bilateral precentral gyri, bilateral superior parietal lobules, and bilateral occipital cortices (Figure 1; Supplementary Table 2).

**Figure 2:**
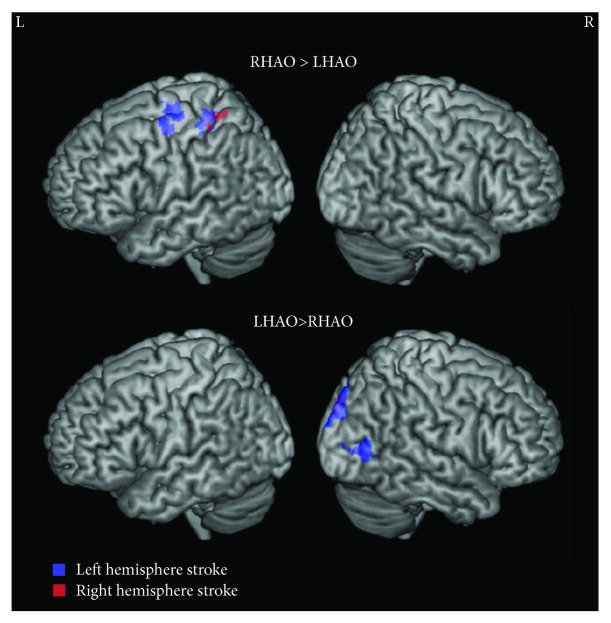
Whole brain activity contrasted between right and left action observation for individuals with stroke. Top: right hand action observation (RHAO) compared to left hand action observation (LHAO), bottom: Left hand action observation (LHAO) compared to right hand action observation (RHAO). Participants with left hemisphere stroke are represented in cool colors (blue); participants with right hemisphere stroke are represented in warm colors (red). Overlap between stroke groups is represented in purple. Thresholded at *Z* > 3.1, corrected for multiple comparisons at *p* < 0.05.

Comparing right and left hand action observation directly revealed the following: *Right* versus *left hand action observation* recruited greater activity in the left postcentral gyrus and superior parietal lobule and the right occipital pole. *Left* versus *right hand action observation* more strongly activated the right occipital pole and intracalcarine cortex (Figure 2; Supplementary Tables 3–4).

#### 3.2.2. Left Hemisphere Stroke Group

Despite our reanalysis using a more stringent threshold, for the left hemisphere stroke group, we find results consistent with the findings reported in Garrison et al. [24]. During *right (corresponding to paretic) hand action observation*, activity was found in the left premotor cortex, bilateral precentral gyri, bilateral supramarginal gyri, and bilateral occipital cortices, among other areas, with greater activity in the left hemisphere (Figure 1; Supplementary Table 1).

During *left (corresponding to nonparetic) hand action observation*, a sparser pattern of activity was found in the left supramarginal gyrus, bilateral precentral gyri, bilateral superior parietal lobules, and bilateral occipital cortices among other areas (Figure 1; Supplementary Table 2).

Comparing right and left hand action observation directly revealed the following: *Right* versus *left hand action observation* recruited greater activity in left precentral gyrus and left postcentral gyrus. *Left* versus *right hand action observation* recruited greater activity in right occipital and occipitotemporal regions (Figure 2; Supplementary Figure S2; Supplementary Tables 3–4).

#### 3.2.3. Nondisabled Control Group

**Figure 3:**
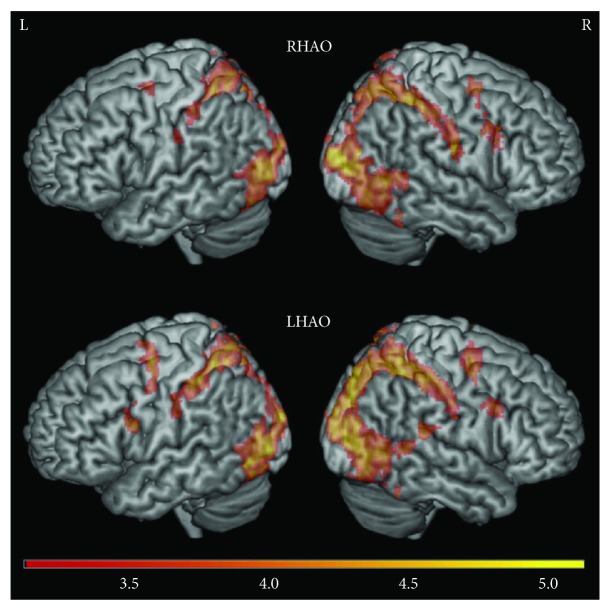
Whole brain activity during right and left action observation for the nondisabled control group. Unlike the two stroke groups, the nondisabled group did not show greater activity on the left hemisphere during right and left hand action observation. Top: right hand action observation (RHAO), bottom: left hand action observation (LHAO). Thresholded at *Z* > 3.1, corrected for multiple comparisons at *p* < 0.05.

Again, consistent with the findings reported in Garrison et al. [24] for the nondisabled control group, during *right (dominant) hand action observation*, activity was found in the right inferior frontal gyrus, right dorsal premotor cortex, right precentral gyrus, bilateral postcentral gyri, bilateral parietal cortices, and bilateral occipital cortices (Figure 3; Supplementary Table 1).

During *left (nondominant) hand action observation*, there was a similar pattern of activity, with activation in the right inferior frontal gyrus, right dorsal premotor cortex, bilateral precentral gyri, bilateral postcentral gyri, bilateral parietal cortices, and bilateral occipital cortices, as well as the right posterior superior temporal sulcus at the temporoparietal junction (Figure 3; Supplementary Table 2).

**Figure 4:**
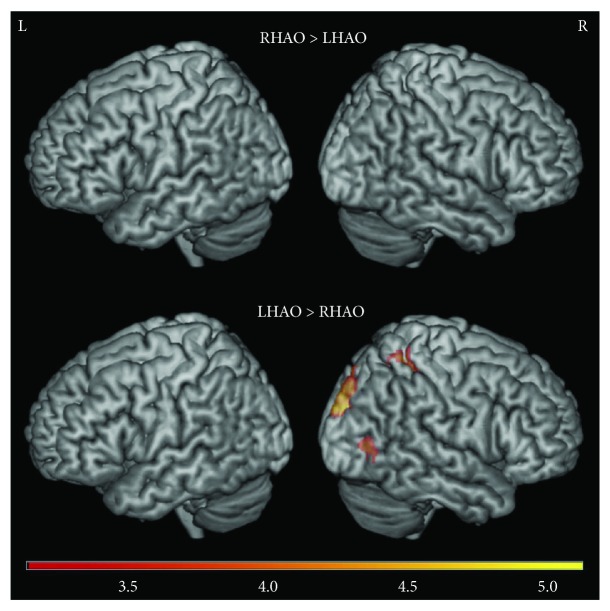
Whole brain activity contrasted between right and left action observation for the nondisabled control group. Top: right hand action observation (RHAO) compared to left hand action observation (LHAO), bottom: left hand action observation (LHAO) compared to right hand action observation (RHAO). Thresholded at *Z* > 3.1, corrected for multiple comparisons at *p* < 0.05.

Comparing right and left hand action observation directly revealed the following: *Right* versus *left hand action observation* revealed no significant activity (Figure 4; Supplementary Table 3). In contrast, *left* versus *right hand action observation* recruited more activity in the right hemisphere, particularly in the right postcentral gyrus, right superior parietal lobule, and right occipital cortex (Figure 4; Supplementary Table 4).

#### 3.2.4. Interim Summary

Whole brain patterns in both stroke groups primarily showed greater left hemisphere activity during *right hand action observation*, while whole brain patterns in nondisabled controls were largely right lateralized during *left hand action observation.* Results here were reported at a relatively stringent threshold of *Z* > 3.1, cluster corrected at *p* < 0.05. Given our smaller group sample sizes and heterogeneity of lesion locations, we also wished to visualize this data at a more lenient threshold (*Z* > 2.3, cluster corrected at *p* < 0.05) to examine whether these laterality trends expanded. At this more lenient threshold, we found the same laterality patterns reported above, but they were extended to much wider regions of the AON (see Supplementary Figure S2).

### 3.3. Laterality Index

**Figure 5:**
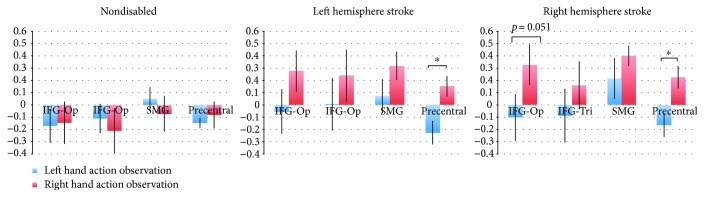
Laterality index in regions of interest. Left: nondisabled participants, middle: participants with left hemisphere stroke, right: participants with right hemisphere stroke; each during left hand (blue) and right hand (red) action observation; in the inferior frontal gyrus pars opercularis (IFG-Op), inferior frontal gyrus pars triangularis (IFG-Tri), supramarginal gyrus (SMG), and precentral gyrus (Precentral). ^∗^ *p* < 0.05. Positive values indicate left hemisphere laterality; negative values indicate right hemisphere laterality.

LI scores range from +1 (all left hemisphere activation only) to −1 (all right hemisphere activation only) and are typically categorized as either bilateral (∣LI∣ ≤ 0.1), hemisphere dominant (0.1 < ∣LI∣ < 0.2), or hemisphere lateralized (∣LI∣ ≥ 0.2; Jansen et al. [37]). Participants in both the right and left hemisphere stroke groups demonstrated a left hemisphere dominant/lateralized pattern of activation across ROIs during *right hand action observation*, independently of which limb was affected by stroke (Figure 5; Supplementary Tables 5–9). For participants with right hemisphere stroke, LI values were as follows: inferior frontal gyrus pars opercularis (LI = 0.33, SEM = 0.17), pars triangularis (LI = 0.16, SEM = 0.19), precentral gyrus (LI = 0.22, SEM = 0.09), and supramarginal gyrus (LI = 0.40, SEM = 0.08). For participants with left hemisphere stroke, LI values were as follows: inferior frontal gyrus pars opercularis (LI = 0.28, SEM = 0.17), pars triangularis (LI = 0.24, SEM = 0.21), precentral gyrus (LI = 0.15, SEM = 0.08), and supramarginal gyrus (LI = 0.32, SEM = 0.12).

Participants in both the right and left hemisphere stroke groups demonstrated a largely bilateral pattern of activation in most ROIs during *left hand action observation*, independent of the limb that was affected by stroke (Figure 5; Supplementary Tables 5–9). Participants with right hemisphere stroke showed bilateral results in the inferior frontal gyrus pars opercularis (LI = −0.10, SEM = 0.19), inferior frontal gyrus pars triangularis (LI = −0.08, SEM = 0.22), right hemisphere lateralization in the precentral gyrus (LI = −0.17, SEM = 0.10), and left hemisphere dominant in the supramarginal gyrus (LI = 0.21, SEM = 0.17). Participants with left hemisphere stroke showed bilateral results in the inferior frontal gyrus pars opercularis (LI = −0.05, SEM = 0.18), pars triangularis (LI = 0.01, SEM = 0.22), and supramarginal gyrus (LI = 0.07, SEM = 0.14) and right hemisphere lateralization in the precentral gyrus (LI = −0.23, SEM = 0.10; Figure 5; Supplementary Tables 5–9). We note that one difference in the LI pattern between stroke groups was that for the right hemisphere stroke group, activity in the supramarginal gyrus was left hemisphere dominant compared to bilateral in the left hemisphere stroke group.

For the nondisabled control group, regions in the AON demonstrated either right hemisphere dominant/lateralized or bilateral activity during both right and left hand action observation (Figure 5; Supplementary Tables 5–9). For *right hand action observation*, LI values are as follows: inferior frontal gyrus pars opercularis (LI = −0.15, SEM = 0.17), pars triangularis (LI = −0.21, SEM = 0.18), precentral gyrus (LI = −0.08, SEM = 0.11), and supramarginal gyrus (LI = −0.07, SEM = 0.14). For *left hand action observation*, LI values are as follows: inferior frontal gyrus pars opercularis (LI = −0.17, SEM = 0.14), pars triangularis (LI = −0.11, SEM = 0.12), precentral gyrus (LI = −0.15, SEM = 0.04), and supramarginal gyrus (LI = 0.05, SEM = 0.09). Importantly, the laterality patterns seen in the nondisabled control group, particularly for right hand action observation, differ from those of the two stroke groups.

### 3.4. Task by Hemisphere by Group Interactions in ROI Activity

#### 3.4.1. Right Hemisphere Stroke Group versus Nondisabled Controls

No three-way interactions were found between group (nondisabled control, right hemisphere stroke), hand observed (right, left), and hemisphere of activity (right, left) for any ROI. A significant two-way interaction was found for group (nondisabled control versus right hemisphere stroke) and hemisphere of activity (right, left) in the supramarginal gyrus (*F*(1, 22) = 7.17, *p* = 0.01, partial *η* ^2^ = 0.25; Bonferroni-corrected *p* value: *p* = 0.04). We then tested for simple main effects. For hemisphere of activity, there was a statistically significant difference between the left and right SMG in the right hemisphere stroke group (*F*(1, 23) = 13.14, *p* = 0.001, partial *η* ^2^ = 0.36), with greater activity in the left compared to right hemisphere (mean ± standard deviation reported for all analyses; left hemisphere: 0.20 ± 0.16, right hemisphere: 0.10 ± 0.19). There were no differences in left and right SMG activity in the ND group (*F*(1, 23) = 2.35, *p* = 0.14). For group, there was a statistically significant difference between groups in the right SMG (*F*(1, 46) = 6.60, *p* = 0.01, partial *η* ^2^ = 0.13), with greater activity for the nondisabled control group compared to the right hemisphere stroke group (nondisabled controls: 0.24 ± 0.19; right hemisphere stroke: 0.10 ± 0.19). No differences in activity were found between ND and RHS groups in the left SMG (*F*(1, 46) = 0.04, *p* = 0.85). Put together, this suggests that the nondisabled control group had more activity in the right compared left supramarginal gyrus, whereas the right hemisphere stroke group had more activity in the left compared to right supramarginal gyrus.

A significant two-way interaction was also found for the hemisphere of activity (right, left) and side of hand observed (right, left) in the precentral gyrus (*F*(1, 22) = 14.78, *p* = 0.001, partial *η* ^2^ = 0.40; Bonferroni-corrected *p* value: *p* = 0.004). We then tested for simple main effects. We did not find a simple main effect for hand observed (i.e., no difference in activity between right and left hand observation for either hemisphere (right hemisphere: *F*(1, 23) = 0.19, *p* = 0.67; left hemisphere: *F*(1, 23) = 3.15, *p* = 0.09)). We found a simple main effect of hemisphere of activity during right hand action observation, with greater activity in the left versus right precentral gyrus (*F*(1, 23) = 7.3, *p* = 0.01; left hemisphere: 0.09 ± 1.5, right hemisphere: 0.04 ± 0.13). Put another way, for both groups, during right hand action observation, activity was greater in the left precentral gyrus.

#### 3.4.2. Right versus Left Hemisphere Stroke Group

We then compared the two stroke groups to one another directly. No three-way interactions were found between the side of the stroke (right, left), hand observed (right, left), and hemisphere of activity (right, left) in any ROI. A significant two-way interaction was found between the hemisphere of activity (right, left) and hand observed (right, left) in the precentral gyrus (*F*(1, 22) = 18.73, *p* < 0.001, partial *η* ^2^ = 0.46; Bonferroni-corrected *p* value: *p* < 0.004); this same interaction was also marginally significant in the inferior frontal gyrus pars opercularis after correcting for multiple comparisons (*F*(1), 22 = 5.74, *p* = 0.025, partial *η* ^2^ = 0.21; Bonferroni-corrected *p* value: *p* = 0.1). We then tested for simple main effects for each interaction. In the precentral gyrus, for effect of hand observed, there was a statistically significant difference in the left precentral gyrus, with greater activity during right hand action observation than during left hand action observation (*F*(1, 23) = 13.78, *p* = 0.001; partial *η* ^2^ = 0.38; left hand action observation: 0.01 ± 0.10, right hand action observation: 0.08 ± 0.11). There was no statistically significant main effect in the right precentral gyrus (*F*(1, 23) = 0.37, *p* = 0.55). For simple main effect of hemisphere of activity, there was a statistically significant difference during right hand action observation (*F*(1, 23) = 11.27, *p* = 0.003, partial *η* ^2^ = 0.33), with greater activity in the left compared to right precentral gyrus (left hemisphere: 0.08 ± 0.11, right hemisphere: 0.03 ± 0.13). There were no significant simple main effects for hemisphere during left hand action observation (*F*(1, 23) = 3.14, *p* = 0.09).

For the inferior frontal gyrus pars opercularis, we found a similar simple main effect for hand observed in the left hemisphere with greater activity during right hand action observation compared to left hand action observation (*F*(1, 23) = 8.1, *p* = 0.009, partial *η* ^2^ = 0.26; right hand action observation: 0.08 ± 0.14, left hand action observation: 0.02 ± 0.14). There were no simple main effects for hand observed in the right hemisphere (*F*(1, 23) = 0.002, *p* = 0.97) and no simple main effects for hemisphere during either right hand action observation (*F*(1, 23) = 2.07, *p* = 0.16) or left hand action observation (*F*(1, 23) = 1.17, *p* = 0.29). Overall, these results suggest there was more left hemisphere activity during *right hand action observation* for both stroke groups. Both stroke groups showed similar hemispheres by hand observed interactions, with brain activity lateralized toward the left motor dominant hemisphere, despite having lesions in different hemispheres.

### 3.5. Lesion Analyses

#### 3.5.1. Lesion Volume Compared between Groups

No significant difference in lesion size was found between the right hemisphere stroke group (*M* = 35593.08 mm^3^, SD = 55942.81) and the left hemisphere stroke group (*M* = 23196.64 mm^3^, SD = 33296.69; *t*(22) = −0.660, *p* = 0.52). This suggests that, despite varying lesion sizes across individuals, reported results were not driven by a difference in overall lesion size between groups. Lesion overlap maps can be found in Supplementary Material Figures S3–S7.

#### 3.5.2. ROI-Lesion Overlap

Overlap between the individual lesions and the AON ROIs (measured as greater than 5% overlap) occurred in only 4 of the individuals in the left hemisphere stroke group and 3 of the individuals in the right hemisphere stroke group. While we had considered also examining the relationship between lesion overlap and laterality index, to examine whether lesion overlap with critical AON regions influenced laterality results, the resulting sample of individuals with lesion overlap was too limited to make an accurate calculation.

### 3.6. Brain Behavior Analyses

Finally, as an exploratory analysis, we examined correlations between ROI activity and motor scores.

#### 3.6.1. Correlations between ROI Activity and WMFT FAS Scores

For participants with right hemisphere stroke, there were no significant correlations between WMFT motor scores and ROI activity. For participants with left hemisphere stroke, nonsignificant trends showing negative correlations between WMFT scores and ROI activity during right hand action observation were found in the inferior frontal gyrus, pars opercularis (*ρ* = −0.51, *p* = 0.091), and pars triangularis (*ρ* = −0.534, *p* = 0.074). In addition, trends in negative correlations between WFMT scores and ROI activity during left hand action observation were found in the right inferior frontal gyrus (*ρ* = −0.545, *p* = 0.067) and precentral gyrus (*ρ* = −0.517, *p* = 0.085). Notably, however, none of these relationships meets the significance threshold after correcting for multiple comparisons (*p* = 0.00625).

#### 3.6.2. Correlations between ROI Activity and Fugl-Meyer Scores

For participants with right hemisphere stroke, no significant correlations were found between Fugl-Meyer scores and ROI activity. For participants with left hemisphere stroke, activity in the right precentral gyrus during right hand action observation demonstrated a trend towards a negative correlation with Fugl-Meyer scores (*ρ* = −0.53, *p* = 0.077), although this again was not significant.

## 4. Discussion

In this study, our primary aim was to examine whether cortical motor activity in the action observation network was lateralized more towards the ipsilesional hemisphere or the motor dominant hemisphere during action observation after a stroke. In both individuals with nondominant right hemisphere stroke and individuals with dominant left hemisphere stroke, AON activity was lateralized toward the left motor dominant hemisphere. There were no significant differences in the lateralization of AON activity between the two stroke groups, despite having lesions in different hemispheres. These results suggest that action observation after stroke may drive greater activity in the motor dominant rather than the ipsilesional hemisphere, at least in our sample of individuals who were right-handed prior to stroke. These findings also differed from our nondisabled control group, in which AON activity was either bilateral or slightly lateralized toward the right nondominant hemisphere.

As mentioned in the Introduction, hemispheric specialization could be an underlying cause of these results in stroke patients. That is, greater AON activity in the dominant left hemisphere may reflect hard-wired properties of the left hemisphere for motor control such as left hemisphere specialization for motor planning, and by extension, action observation, compared to the right hemisphere, regardless of which hemisphere is affected after stroke. This may mean that the motor dominant hemisphere may also play a role in the effectiveness of action observation therapy. While not tested here, it is possible that driving activity in the motor dominant hemisphere via action observation could help to promote motor recovery after stroke. Future studies could examine whether dominant hemisphere activation during action observation relates to changes in motor recovery following poststroke action observation therapy.

In addition, previous work has shown that the motor dominant hemisphere has greater descending motor pathways than the nondominant hemisphere has [42, 43]. Additionally, the typically motor dominant left precentral gyrus receives inputs from both the contralateral and ipsilateral hand, whereas the nondominant right precentral gyrus receives the majority of inputs solely from the contralateral left hand [44]. After a stroke, this imbalance in motor pathways may be accentuated to more strongly engage left-lateralized activity during right hand action observation and bilateral activity during left hand action observation. Likewise, individuals with dominant left hemisphere stroke have been shown to experience some motor deficits in both hands, whereas those with right hemisphere stroke typically only experience motor deficits in the contralateral left hand [45]. Again, while it remains to be tested in a future study, it is possible that individuals with nondominant right hemisphere stroke are able to continue to use their dominant (nonparetic) hand, and individuals with dominant left hemisphere stroke may place more emphasis on using their dominant (paretic) hand in spite of its impairments. Therefore, both groups may place greater emphasis on the dominant hand when asked to observe and later imitate actions, explaining the greater activation in the dominant left hemisphere in both groups.

Based on this logic, we might expect individuals with nondominant hand paresis to experience poorer motor recovery due to the ability to rely on the nonparetic dominant hand. Although results across studies vary, there is indeed evidence that individuals with right hemisphere stroke show poorer motor recovery of the affected nondominant left hand than those with left hemisphere stroke and an affected dominant right hand [46, 47]. Related, a limitation of the current study is the fact that the nondominant right hemisphere stroke group also had greater motor impairments (lower Fugl-Meyer and Wolf Motor Function Test scores) of the affected hand than the dominant left hemisphere stroke group, despite both groups falling within the eligibility range of moderate-to-severe motor impairments and having no differences in lesion volumes. While this may be reflective of trends in the general stroke population, this difference does introduce a potential confound, as the level of impairment could also drive patterns of cortical activity. However, importantly, there was no significant relationship between the level of impairment and brain activity within the right hemisphere stroke group, suggesting that the level of impairment for individuals with right hemisphere stroke does not influence AON activity. Given the small sample size, we further visually inspected the subject-level correlation data between brain activity and motor impairment, in case there were potential trends that were not significant. However, there were no relationships or trends between level of motor impairment and brain activity across this sample, such that individuals with less severe stroke did not show any differences in laterality index than individuals with more severe stroke. This suggests that between-group differences in level of motor impairment did not drive these left-lateralized results. Regardless, further research with a larger sample of nondominant, right hemisphere stroke patients, with a wider range from mild to severe impairments, is needed to confirm these findings.

In addition, although both right and left hemisphere stroke groups had *stronger* activations in the left hemisphere during action observation, it should be noted that there was still significant activity observed in the right hemisphere in all groups, including the two stroke groups (see Figure 1; Tables S1 and S2). General whole brain activity during action observation was bilateral for all groups, and the lateralization results emerged primarily when examining the laterality index, which calculates a ratio of left to right hemisphere activity. Thus, while we emphasize the role of the dominant left hemisphere because AON activity examined using the laterality index calculation was lateralized to the dominant left hemisphere more in both stroke groups compared to the control group, there is likely also a role of right hemisphere activity for all groups during action observation.

Finally, in line with this, we note that the healthy, age-matched control group showed bilateral or slightly right-lateralized activity. Although this is in line with many previous studies showing that AON activity in healthy right-handed individuals is typically bilateral [35, 48, 49], a previous study specifically examining the laterality index in healthy individuals showed left-lateralized AON patterns [50]. In reconciling our current findings with the previous literature, we first note that in that study, the laterality index was performed on entire lobes (e.g., LI of the frontal lobe was left-lateralized) whereas here we calculated the LI within specific AON nodes. This specificity may have affected results. In addition, a primary factor that may contribute to these disparate results is age. The previous study examining the laterality index of the AON in healthy individuals used healthy younger adults, while in our study, we used healthy older adults (age-matched to our stroke population). Research has shown that older adults typically recruit additional and broader regions of the AON compared to younger adults [51–54]. While further research is needed to specifically examine the laterality of the AON in healthy younger versus older adults, it is possible that our healthy older adult control group shows more bilateral or slightly right-lateralized AON activity, instead of left-lateralized AON activity, due to age-related changes in the AON.

### 4.1. Limitations and Future Directions

Our results support the idea that hemispheric dominance affects patterns of neural activity induced by action observation after stroke. While the current sample size was small (12 participants per group), both right and left hemisphere stroke groups (24 participants in total) showed similar patterns of left-lateralized AON activity during observation of right hand actions and bilateral AON activity during observation of left hand actions, which was different from nondisabled individuals. However, as noted in the Discussion, the functional abilities of the two groups were significantly different. Notably, we did not find a relationship between the level of impairment and lateralization of AON activity within the right hemisphere group, suggesting that the differing functional levels were not associated with different brain activation patterns. However, we acknowledge that this group difference still provides a possible confounding factor as previous work has shown that degree of motor severity influences cortical recruitment [55]. In addition, previous work has shown that patients with greater corticospinal tract (CST) damage also show greater recruitment of cortical areas [56, 57]. Although lesion volumes were similar between groups, the current study did not specifically examine overlap of the lesion with the CST. Thus, a replication of these patterns in a larger, more diverse sample, with individuals across a range of motor impairment levels (mild, moderate, severe), and examining the overlap of the lesion with the CST, would improve our understanding of how the current findings relate to a diverse population of individuals after stroke.

Our findings support a possible specialization of the motor dominant hemisphere during action observation following stroke. However, the functional implications of this activation are unclear. An important question is whether and how these results, and recovery from nondominant (right hemisphere) stroke, may relate to real-world hand usage. Future studies might examine real-world hand usage during daily activities (e.g., using accelerometers [58, 59]), and relate that to laterality patterns in brain activity following stroke.

In addition, here we showed that action observation engages the motor dominant hemisphere in individuals who are right hemisphere dominant (left-handed) prior to stroke. Right hemisphere dominance is less common, and therefore, the population with stroke will be smaller and less is known about motor control in this group. However, given our findings' interpretations, we might expect AON activity to be lateralized toward the dominant right hemisphere in that group. A more complete understanding of the relationship between motor dominance, hemisphere of stroke, and AON activity should be studied to enable personalized interventions in stroke neurorehabilitation.

Finally, given the conventional wisdom that activity in the ipsilesional hemisphere promotes recovery of motor function after stroke [2–4], a logical next step is to evaluate what our findings may mean for stroke rehabilitation. Our findings, and those of others, suggest that optimal recovery of motor function may depend on the hemisphere of the lesion [20]. As such, parameters of AOT, such as whether individuals with stroke observe actions corresponding to their paretic limb only, versus observation of bilateral movements, may yield different results for different participants. While few stroke neuroimaging studies have been adequately powered to compare between right and left hemisphere stroke groups, it may be a critical difference that affects stroke rehabilitation and motor recovery. Future large-scale studies should examine whether and how the hemispheric dominance of the lesioned hemisphere affects neural activity during different types of therapy and subsequent motor recovery.

## Figures and Tables

**Table 1:** Demographics of participants. Fugl-Meyer Assessment, Upper Extremity (FMA-UE; out of 66 points) and Wolf Motor Function Test (WMFT; out of 5 points). Stroke location was characterized as either internal capsule only (IC) or internal capsule plus cerebral cortex (C + IC). “--” indicates missing values.

Subject number	FMA-UE	WMFT	Age (years)	Sex	Time since stroke (months)	Location
Right hemisphere stroke						
1	5	1	66	F	80	IC
2	17	1	65	F	22	IC
3	10	1	70	M	202	C + IC
4	8	1	59	F	46	C + IC
5	14	—	79	M	168	C + IC
6	16	2	56	F	6	IC
7	18	1.5	52	M	67	IC
8	31	1	33	M	10	C + IC
9	5	0	61	M	118	C + IC
10	31	3.25	71	M	74	IC
11	5	0	65	M	48	IC
12	18	1.25	35	F	21	IC
Mean	**14.83**	**1.18**	**59.33**	**5** F	**71.83**	**7 IC**
SDEV	9.09	0.90	13.81		62.43	

Left hemisphere stroke						
1	48	3.33	64	F	60	IC
2	13	0.5	64	F	180	C + IC
3	46	3.25	55	M	48	IC
4	18	2	74	M	204	IC
5	40	2	39	M	24	IC
6	13	0.67	73	M	48	IC
7	31	2.5	85	F	96	IC
8	14	0.25	51	F	72	C + IC
9	47	3.33	74	F	108	C + IC
10	15	0.75	68	F	72	C + IC
11	37	2.5	71	M	96	C + IC
12	35	4	71	M	48	C + IC
Mean	**29.75**	**2.09**	**65.75**	**6** F	**88.00**	**6 IC**
SDEV	14.29	1.28	12.34		54.47	

## Data Availability

The authors are happy to share their data. However, due to IRB regulations, the authors may share only deidentified subsets of the data from this study upon request.

## Supplementary Materials

Supplementary Materials Figure S1: Example from video stimuli during fMRI. Figure S2: Whole brain activity contrasted between right and left action observation compared between stroke and control groups at a more lenient threshold. Figure S3: Lesion overlap heat map (whole group). Figure S4: Lesion overlap heat map for cortical left hemisphere strokes (*n* = 6). Figure S5: Lesion overlap heat map for subcortical left hemisphere strokes (*n* = 6). Figure S6: Lesion overlap heat map for cortical right hemisphere stroke (*n* = 5). Figure S7: Lesion overlap heat map for subcortical right hemisphere stroke (*n* = 7). Table S1: Main effect of right hand action observation. Table S2: Main effect of left hand action observation. Table S3: Brain activity during right hand action observation versus left hand action observation (RHAO > LHAO). Table S4: Brain activity during left hand action observation versus right hand action observation (LHAO > RHAO). Table S5: Group laterality index values. Table S6: Individual laterality index values—inferior frontal gyrus and pars opercularis. Table S7: Individual laterality index values—inferior frontal gyrus and pars triangularis. Table S8: Individual laterality index values—supramarginal gyrus. Table S9: Individual laterality index values—precentral gyrus.

## References

[1] Mozaffarian D., Benjamin E. J., Go A. S. (2016). Heart disease and stroke statistics-2016 update a report from the American Heart Association. *Circulation*.

[2] Marshall R. S., Perera G. M., Lazar R. M., Krakauer J. W., Constantine R. C., DeLaPaz R. L. (2000). Evolution of cortical activation during recovery from corticospinal tract infarction. *Stroke*.

[3] Nudo R. J. (2006). Mechanisms for recovery of motor function following cortical damage. *Current Opinion in Neurobiology*.

[4] Ward N. S., Cohen L. G. (2004). Mechanisms underlying recovery of motor function after stroke. *Archives of Neurology*.

[5] Ward N. S., Brown M. M., Thompson A. J., Frackowiak R. S. J. (2003). Neural correlates of outcome after stroke: a cross-sectional fMRI study. *Brain*.

[6] Werhahn K. J., Conforto A. B., Kadom N., Hallett M., Cohen L. G. (2003). Contribution of the ipsilateral motor cortex to recovery after chronic stroke. *Annals of Neurology*.

[7] Fregni F., Boggio P. S., Mansur C. G. (2005). Transcranial direct current stimulation of the unaffected hemisphere in stroke patients. *Neuroreport*.

[8] Liew S.-L., Santarnecchi E., Buch E. R., Cohen L. G. (2014). Non-invasive brain stimulation in neurorehabilitation: local and distant effects for motor recovery. *Frontiers in Human Neuroscience*.

[9] Lindenberg R., Renga V., Zhu L. L., Nair D., Schlaug G. (2010). Bihemispheric brain stimulation facilitates motor recovery in chronic stroke patients. *Neurology*.

[10] Takeuchi N., Chuma T., Matsuo Y., Watanabe I., Ikoma K. (2005). Repetitive transcranial magnetic stimulation of contralesional primary motor cortex improves hand function after stroke. *Stroke*.

[11] Mani S., Mutha P. K., Przybyla A., Haaland K. Y., Good D. C., Sainburg R. L. (2013). Contralesional motor deficits after unilateral stroke reflect hemisphere-specific control mechanisms. *Brain*.

[12] Sainburg R. L., Duff S. V. (2006). Does motor lateralization have implications for stroke rehabilitation?. *Journal of Rehabilitation Research and Development*.

[13] Schaefer S. Y., Haaland K. Y., Sainburg R. L. (2007). Ipsilesional motor deficits following stroke reflect hemispheric specializations for movement control. *Brain*.

[14] Winstein C. J., Pohl P. S. (1995). Effects of unilateral brain damage on the control of goal-directed hand movements. *Experimental Brain Research*.

[15] Yadav V., Sainburg R. L. (2014). Limb dominance results from asymmetries in predictive and impedance control mechanisms. *PLoS One*.

[16] Rizzolatti G., Craighero L. (2004). The mirror-neuron system. *Annual Review of Neuroscience*.

[17] Celnik P., Webster B., Glasser D. M., Cohen L. G. (2008). Effects of action observation on physical training after stroke. *Stroke*.

[18] Ertelt D., Small S., Solodkin A. (2007). Action observation has a positive impact on rehabilitation of motor deficits after stroke. *NeuroImage*.

[19] Franceschini M., Ceravolo M. G., Agosti M. (2012). Clinical relevance of action observation in upper-limb stroke rehabilitation: a possible role in recovery of functional dexterity. A randomized clinical trial. *Neurorehabilitation and Neural Repair*.

[20] Sale P., Ceravolo M. G., Franceschini M. (2014). Action observation therapy in the subacute phase promotes dexterity recovery in right-hemisphere stroke patients. *BioMed Research International*.

[21] Sarasso S., Määttä S., Ferrarelli F., Poryazova R., Tononi G., Small S. L. (2014). Plastic changes following imitation-based speech and language therapy for aphasia: a high-density sleep EEG study. *Neurorehabilitation and Neural Repair*.

[22] Sugg K., Müller S., Winstein C., Hathorn D., Dempsey A. (2015). Does Action Observation Training with Immediate Physical Practice Improve Hemiparetic Upper-Limb Function in Chronic Stroke?. *Neurorehabilitation and Neural Repair*.

[23] Garrison K. A., Winstein C. J., Aziz-Zadeh L. (2010). The mirror neuron system: a neural substrate for methods in stroke rehabilitation. *Neurorehabilitation and Neural Repair*.

[24] Garrison K. A., Aziz-Zadeh L., Wong S. W., Liew S. L., Winstein C. J. (2013). Modulating the motor system by action observation after stroke. *Stroke*.

[25] Oldfield R. C. (1971). The assessment and analysis of handedness: the Edinburgh inventory. *Neuropsychologia*.

[26] Wolf S. L., Catlin P. A., Ellis M., Archer A. L., Morgan B., Piacentino A. (2001). Assessing Wolf motor function test as outcome measure for research in patients after stroke. *Stroke*.

[27] Jenkinson M., Bannister P., Brady M., Smith S. (2002). Improved optimization for the robust and accurate linear registration and motion correction of brain images. *NeuroImage*.

[28] Jenkinson M., Smith S. (2001). A global optimisation method for robust affine registration of brain images. *Medical Image Analysis*.

[29] Smith S. M. (2002). Fast robust automated brain extraction. *Human Brain Mapping*.

[30] Woolrich M. W., Ripley B. D., Brady M., Smith S. M. (2001). Temporal autocorrelation in univariate linear modeling of FMRI data. *NeuroImage*.

[31] Worsley K. J. (2001). Statistical analysis of activation images. *Functional Magnetic Resonance Imaging*.

[32] Beckmann C. F., Jenkinson M., Smith S. M. (2003). General multilevel linear modeling for group analysis in FMRI. *NeuroImage*.

[33] Woolrich M. (2008). Robust group analysis using outlier inference. *NeuroImage*.

[34] Woolrich M. W., Behrens T. E. J., Beckmann C. F., Jenkinson M., Smith S. M. (2004). Multilevel linear modelling for FMRI group analysis using Bayesian inference. *NeuroImage*.

[35] Caspers S., Zilles K., Laird A. R., Eickhoff S. B. (2010). ALE meta-analysis of action observation and imitation in the human brain. *NeuroImage*.

[36] Ito K. L., Liew S.-L. (2016). Calculating the laterality index using FSL for stroke neuroimaging data. *GigaScience*.

[37] Jansen A., Menke R., Sommer J. (2006). The assessment of hemispheric lateralization in functional MRI-robustness and reproducibility. *NeuroImage*.

[38] Riley J. D., le V., der-Yeghiaian L. (2011). Anatomy of stroke injury predicts gains from therapy. *Stroke*.

[39] Liew S.-L., Anglin J. M., Banks N. W. (2018). A large, open source dataset of stroke anatomical brain images and manual lesion segmentations. *Scientific Data*.

[40] Rorden C., Brett M. (2000). Stereotaxic display of brain lesions. *Behavioural Neurology*.

[41] Fugl-Meyer A. R., Jääskö L., Leyman I., Olsson S., Steglind S. (1975). The post-stroke hemiplegic patient. 1. A method for evaluation of physical performance. *Scandinavian Journal of Rehabilitation Medicine*.

[42] Hammond G. (2002). Correlates of human handedness in primary motor cortex: a review and hypothesis. *Neuroscience & Biobehavioral Reviews*.

[43] Rademacher J., Bürgel U., Geyer S. (2001). Variability and asymmetry in the human precentral motor system: a cytoarchitectonic and myeloarchitectonic brain mapping study. *Brain*.

[44] Kim S., Ashe J., Hendrich K. (1993). Functional magnetic resonance imaging of motor cortex: hemispheric asymmetry and handedness. *Science*.

[45] Haaland K. Y., Harrington D. L. (1996). Hemispheric asymmetry of movement. *Current Opinion in Neurobiology*.

[46] Harris J. E., Eng J. J. (2006). Individuals with the dominant hand affected following stroke demonstrate less impairment than those with the nondominant hand affected. *Neurorehabilitation and Neural Repair*.

[47] Ween J. E., Alexander M. P., D'Esposito M., Roberts M. (1996). Factors predictive of stroke outcome in a rehabilitation setting. *Neurology*.

[48] Aziz-Zadeh L., Koski L., Zaidel E., Mazziotta J., Iacoboni M. (2006). Lateralization of the human mirror neuron system. *Journal of Neuroscience*.

[49] Aziz-Zadeh L., Maeda F., Zaidel E., Mazziotta J., Iacoboni M. (2002). Lateralization in motor facilitation during action observation: a TMS study. *Experimental Brain Research*.

[50] Cabinio M., Blasi V., Borroni P. (2010). The shape of motor resonance: right- or left-handed?. *NeuroImage*.

[51] Costello M. C., Bloesch E. K. (2017). Are older adults less embodied? A review of age effects through the lens of embodied cognition. *Frontiers in Psychology*.

[52] Diersch N., Mueller K., Cross E. S., Stadler W., Rieger M., Schütz-Bosbach S. (2013). Action prediction in younger versus older adults: neural correlates of motor familiarity. *PLoS One*.

[53] Kuehn E., Perez-Lopez M. B., Diersch N., Döhler J., Wolbers T., Riemer M. (2017). Embodiment in the aging mind. *Neuroscience & Biobehavioral Reviews*.

[54] Léonard G., Tremblay F. (2007). Corticomotor facilitation associated with observation, imagery and imitation of hand actions: a comparative study in young and old adults. *Experimental Brain Research*.

[55] Rehme A. K., Fink G. R., von Cramon D. Y., Grefkes C. (2010). The role of the contralesional motor cortex for motor recovery in the early days after stroke assessed with longitudinal FMRI. *Cerebral Cortex*.

[56] Ward N. S., Newton J. M., Swayne O. B. C. (2007). The relationship between brain activity and peak grip force is modulated by corticospinal system integrity after subcortical stroke. *European Journal of Neuroscience*.

[57] Ward N. S., Newton J. M., Swayne O. B. C. (2006). Motor system activation after subcortical stroke depends on corticospinal system integrity. *Brain*.

[58] Bailey R. R., Klaesner J. W., Lang C. E. (2014). An accelerometry-based methodology for assessment of real-world bilateral upper extremity activity. *PLoS One*.

[59] Lang C. E., Wagner J. M., Edwards D. F., Dromerick A. W. (2007). Upper extremity use in people with hemiparesis in the first few weeks after stroke. *Journal of Neurologic Physical Therapy*.

