# Supplementary material for: Laterality of Poststroke Cortical Motor Activity during Action Observation Is Related to Hemispheric Dominance
Source: Neural Plast. 2018 May 28;2018:3524960. doi: 10.1155/2018/3524960 (PMC5994588; doi:10.1155/2018/3524960)
Supplement: Supplementary Materials — Figure S1: Example from video stimuli during fMRI. Figure S2: Whole brain activity contrasted between right and left action observation compared between stroke and control groups at a more lenient threshold. Figure S3: Lesion overlap heat map (whole group). Figure S4: Lesion overlap heat map for cortical left hemisphere strokes (n = 6). Figure S5: Lesion overlap heat map for subcortical left hemisphere strokes (n = 6). Figure S6: Lesion overlap heat map for cortical right hemisphere stroke (n = 5). Figure S7: Lesion overlap heat map for subcortical right hemisphere stroke (n = 7). Table S1: Main effect of right hand action observation. Table S2: Main effect of left hand action observation. Table S3: Brain activity during right hand action observation versus left hand action observation (RHAO > LHAO). Table S4: Brain activity during left hand action observation versus right hand action observation (LHAO > RHAO). Table S5: Group laterality index values. Table S6: Individual laterality index values—inferior frontal gyrus and pars opercularis. Table S7: Individual laterality index values—inferior frontal gyrus and pars triangularis. Table S8: Individual laterality index values—supramarginal gyrus. Table S9: Individual laterality index values—precentral gyrus. [file 3524960.f1.docx]

**SUPPLEMENTAL FIGURES**

**
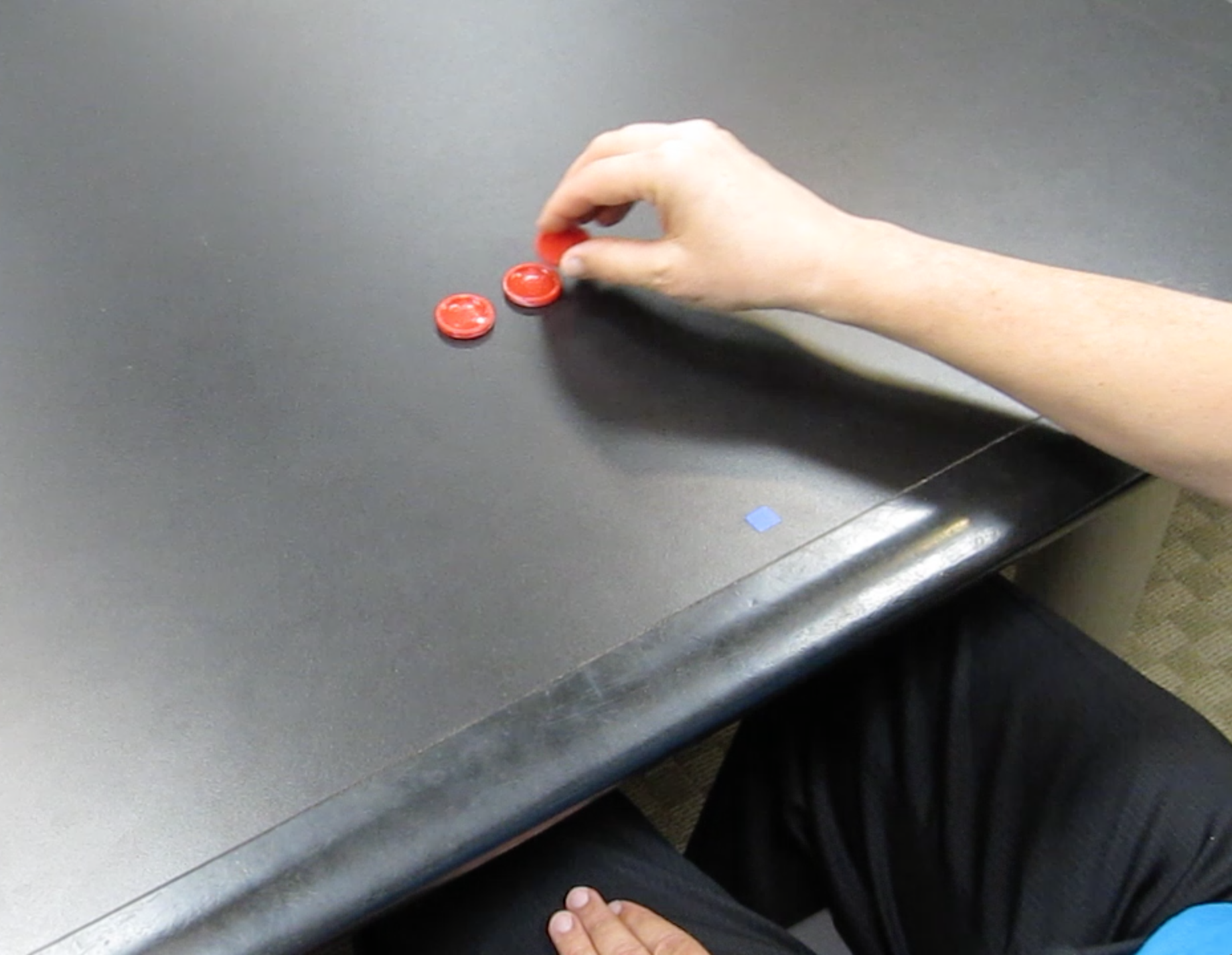
**

**Figure S1. Example from video stimuli during fMRI.** Video stimuli during the fMRI experiment included actions adapted from the Wolf Motor Function Test and included picking up a pencil, picking up a paperclip, stacking checkers, and flipping cards. Each action was performed by the left and right hands. This figure shows an example still image from the stacking checkers video with the right hand.


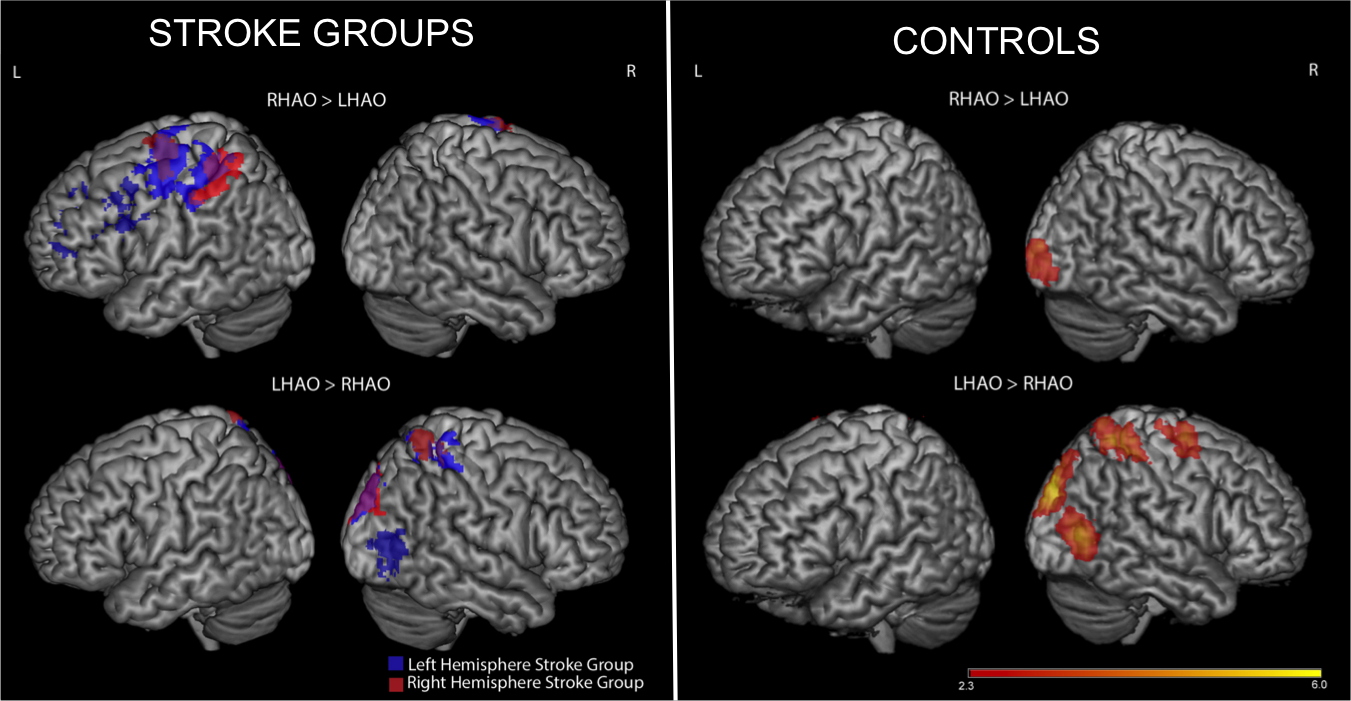


**Figure S2. Whole brain activity contrasted between right and left action observation compared between stroke and control groups at a more lenient threshold. LEFT: Stroke groups: Top:** Right hand action observation (RHAO) compared to left hand action observation (LHAO), **Bottom:** Left hand action observation (LHAO) compared to right hand action observation (RHAO). Participants with left hemisphere stroke are represented in cool colors (blue); participants with right hemisphere stroke are represented in warm colors (red). Overlap between stroke groups is represented in purple. **RIGHT: Nondisabled controls: Top:** Right hand action observation (RHAO) compared to left hand action observation (LHAO), **Bottom:** Left hand action observation (LHAO) compared to right hand action observation (RHAO).

Thresholded at Z>2.3, corrected for multiple comparisons at p<0.05.

**
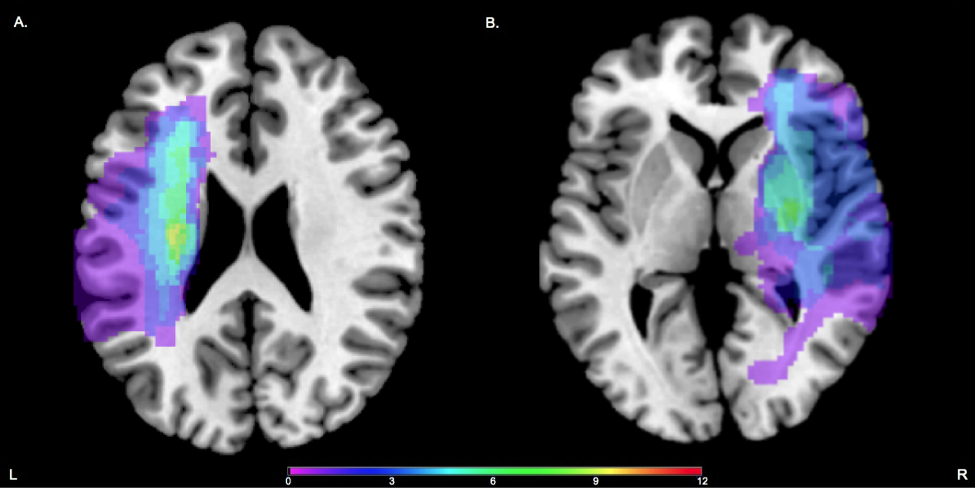
**

**Figure S3. Lesion overlap heat map**. A. Lesions in the left hemisphere stroke group (n=12); B. Lesions in the right hemisphere stroke group (n=12).


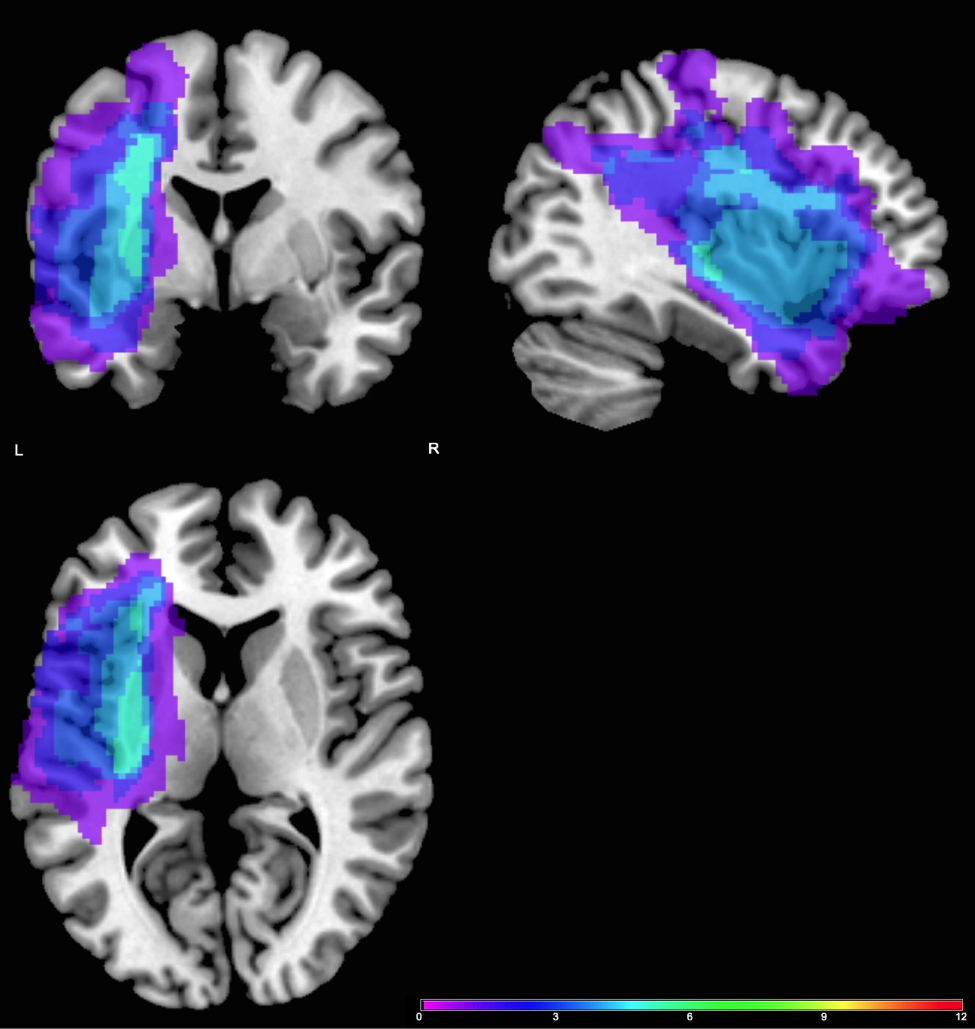


**Figure S4. Lesion overlap heat map for cortical left hemisphere strokes** (n=6)**.**

**
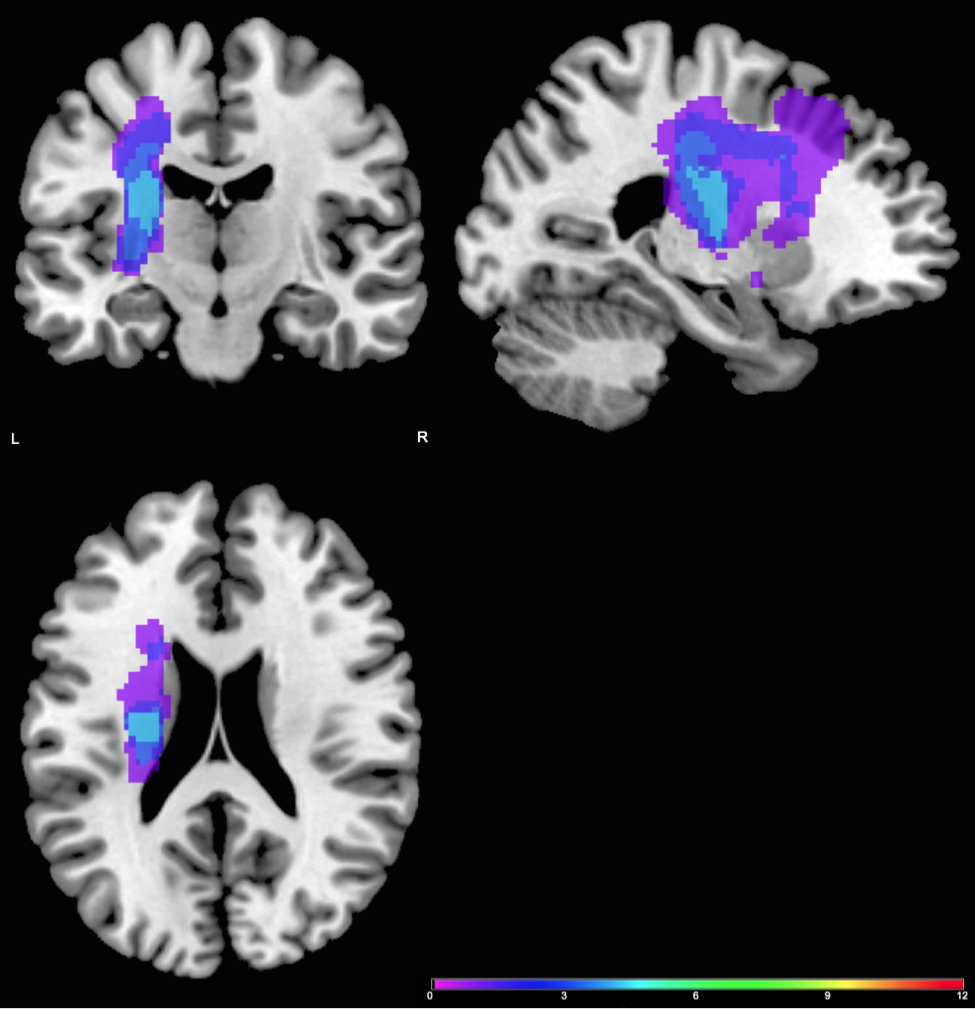
**

**Figure S5. Lesion overlap heat map for subcortical left hemisphere strokes** (n=6).

**
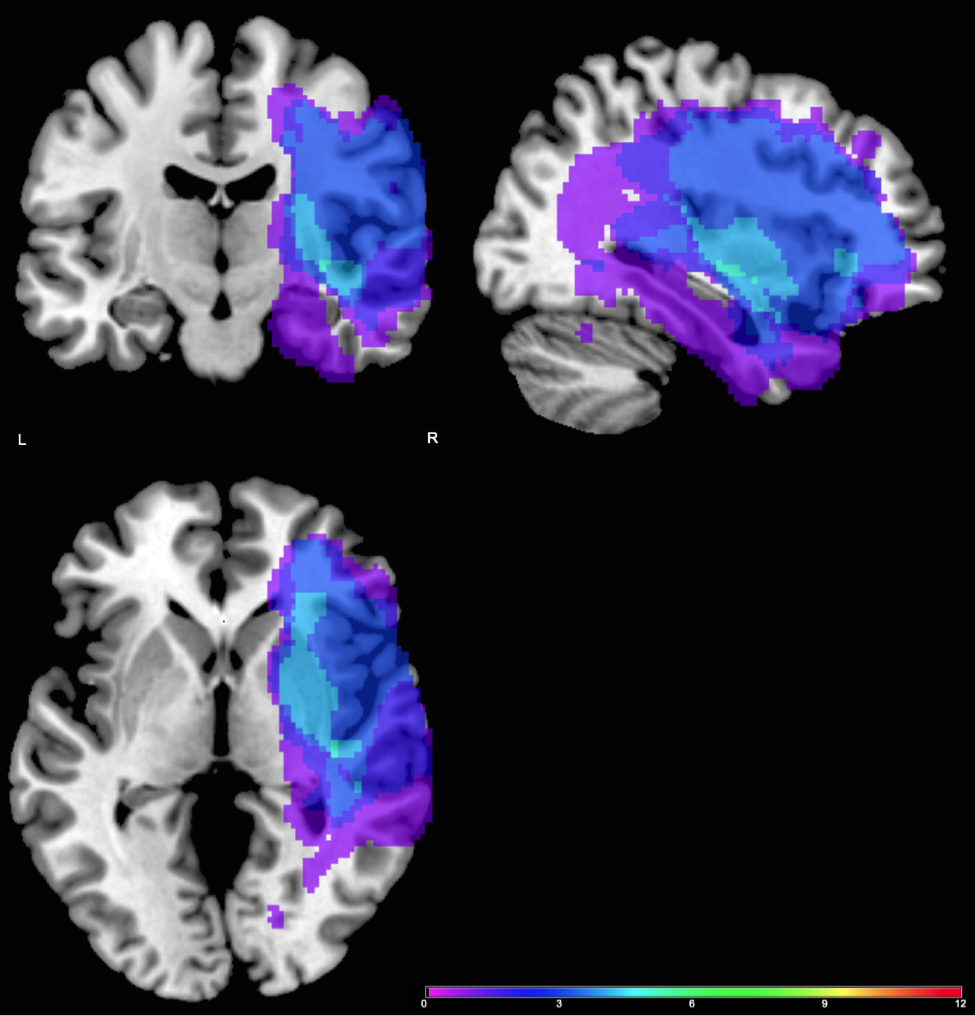
**

**Figure S6. Lesion overlap heat map for cortical right hemisphere stroke** (n=5).

**
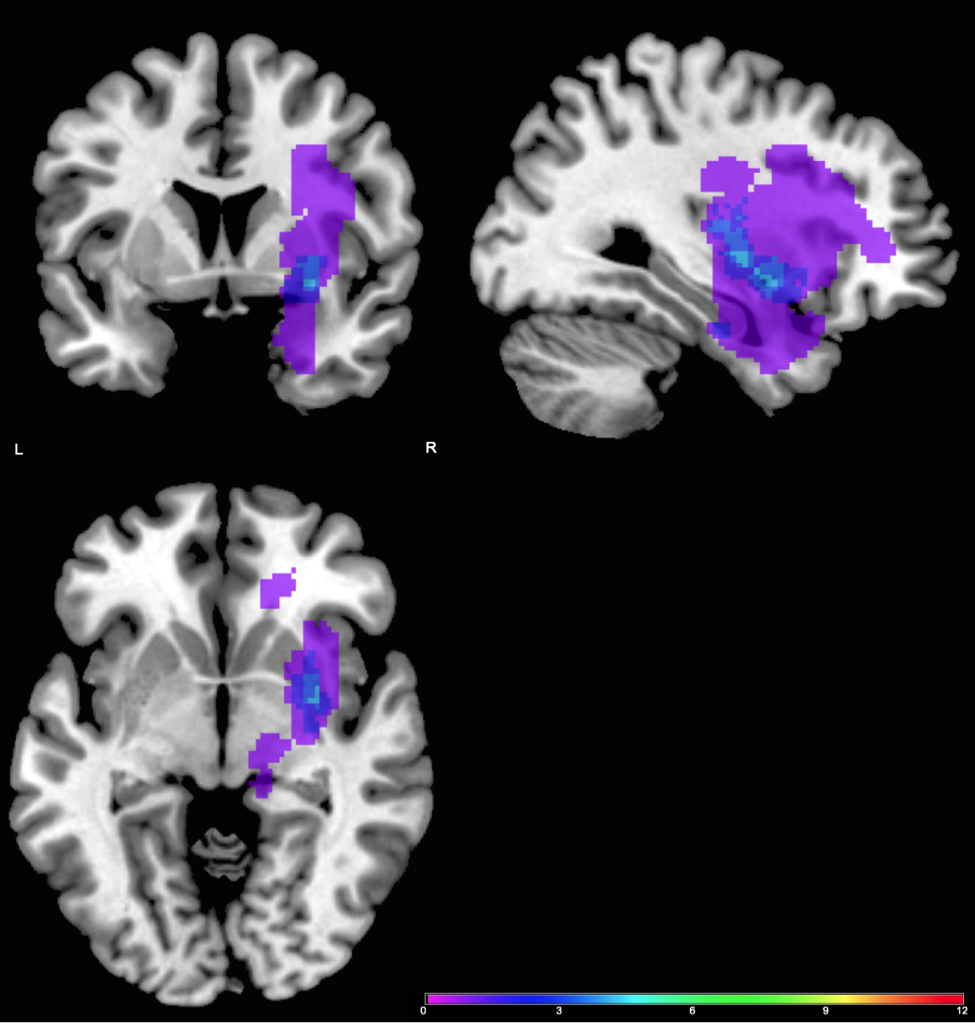
**

**Figure S7. Lesion overlap heat map for subcortical right hemisphere stroke** (n=7).

**Table S1. Main effect of right hand action observation.** Peak activations during right hand action observation for participants with right hemisphere stroke, participants with left hemisphere stroke, and non-disabled participants.

| BRAIN REGION | BA | SIDE | Z | | x | | y | | z | |  |
| --- | --- | --- | --- | --- | --- | --- | --- | --- | --- | --- | --- |
| RIGHT HEMISPHERE STROKE |  |  |  | |  | |  | |  | |  |
| Occipital Fusiform Gyrus | 37 | L | 5.29 | | -14 | | -90 | | -12 | |  |
| Lateral Occipital Cortex, inferior division | 19 | L | 5.05 | | -42 | | -80 | | -6 | |  |
| Lateral Occipital Cortex, superior division | 19 | R | 5.01 | | 28 | | -76 | | 38 | |  |
| Superior Parietal Lobule | 5 | L | 4.98 | | -32 | | -40 | | 48 | |  |
| Middle Temporal Gyrus, temporooccipital part | 21 | R | 4.83 | | 56 | | -60 | | 6 | |  |
| Lateral Occipital Cortex, superior division | 19 | L | 4.75 | | -26 | | -64 | | 58 | |  |
| Lateral Occipital Cortex, inferior division | 19 | R | 4.59 | | 48 | | -66 | | -2 | |  |
| Occipital Pole | 17 | R | 4.57 | | 20 | | -92 | | 10 | |  |
| Superior Parietal Lobule | 5 | R | 4.52 | | 32 | | -42 | | 46 | |  |
| Postcentral Gyrus | 1, 2, 3 | L | 4.5 | | -36 | | -36 | | 46 | |  |
| Occipital Pole | 17 | L | 4.49 | | -22 | | -92 | | 8 | |  |
| Supramarginal Gyrus, anterior division | 40 | L | 4.45 | | -54 | | -32 | | 32 | |  |
| Precentral Gyrus | 4 | L | 4.32 | | -34 | | -8 | | 66 | |  |
| Superior Frontal Gyrus | 6 | L | 4.13 | | -24 | | -2 | | 66 | |  |
| LEFT HEMISPHERE STROKE |  |  |  | |  | |  | |  | |  |
| Occipital Fusiform Gyrus | 37 | R | 6.21 | | 30 | | -88 | | -12 | |  |
| Lingual Gyrus | 19 | L | 6.04 | | -8 | | -84 | | -4 | |  |
| Lateral Occipital Cortex, inferior division | 19 | L | 5.95 | | -42 | | -78 | | 2 | |  |
| Precentral Gyrus | 4 | L | 5.69 | | -42 | | -6 | | 62 | |  |
| Lateral Occipital Cortex, superior division | 19 | L | 5.52 | | -26 | | -62 | | 48 | |  |
| Occipital Pole | 17 | R | 5.51 | | 26 | | -90 | | -10 | |  |
| Superior Parietal Lobule | 5 | R | 4.95 | | 34 | | -50 | | 60 | |  |
| Superior Frontal Gyrus | 6 | L | 4.82 | | -26 | | -6 | | 70 | |  |
| Precentral Gyrus | 4 | R | 4.76 | | 34 | | -6 | | 68 | |  |
| Middle Frontal Gyrus | 46 | L | 4.58 | | -46 | | 8 | | 32 | |  |
| Lateral Occipital Cortex, superior division | 19 | R | 4.44 | | 22 | | -70 | | 46 | |  |
| Middle Frontal Gyrus | 46 | R | 3.7 | | 36 | | 2 | | 46 | |  |
| Occipital Fusiform Gyrus | 37 | R | 6.21 | | 30 | | -88 | | -12 | |  |
| Lingual Gyrus | 19 | L | 6.04 | | -8 | | -84 | | -4 | |  |
| NON-DISABLED CONTROLS |  |  |  | |  | |  | |  | |  |
| Occipital Fusiform Gyrus | 37 | L | 5.52 | | -28 | | -68 | | -12 | |  |
| Lateral Occipital Cortex, inferior division | 19 | L | 5.07 | | -36 | | -80 | | -12 | |  |
| Superior Parietal Lobule | 5 | R | 4.98 | | 34 | | -46 | | 52 | |  |
| Occipital Pole | 17 | R | 4.96 | | 28 | | -94 | | 12 | |  |
| Lateral Occipital Cortex, superior division | 19 | R | 4.93 | | 40 | | -88 | | 8 | |  |
| Inferior Temporal Gyrus, temporooccipital part | 20 | R | 4.9 | | 44 | | -60 | | -12 | |  |
| Temporal Occipital Fusiform Cortex | 20 | L | 4.68 | | -40 | | -60 | | -10 | |  |
| Middle Frontal Gyrus | 46 | R | 4.52 | | 34 | | -2 | | 54 | |  |
| Lateral Occipital Cortex, superior division | 19 | L | 4.48 | | -20 | | -66 | | 56 | |  |
| Inferior Frontal Gyrus, pars opercularis | 44 | R | 4.3 | | 48 | | 12 | | 28 | |  |
| Precentral Gyrus | 4 | L | 4.12 | | -26 | | -8 | | 56 | |  |
| Superior Frontal Gyrus | 6 | R | 3.87 | | 24 | | -2 | | 62 | |  |
| Middle Frontal Gyrus | 46 | L | 3.83 | | -30 | | -4 | | 62 | |  |
| Precentral Gyrus | 4 | R | 3.54 | | 34 | | -2 | | 68 | |  |
| Superior Frontal Gyrus | 6 | L | 3.53 | | -14 | | -2 | | 62 | |  |
| Juxtapositional Lobule Cortex | 6 | L | 3.15 | | -10 | | 0 | | 62 | |  |
| MNI coordinates, Z>3.1; cluster threshold *p*<0.05 | | | |  | |  | |  | |  | |

**Table S2.** **Main effect of left hand action observation.** Regions activated during left hand action observation for participants with right hemisphere stroke, participants with left hemisphere stroke, and nondisabled controls.

| BRAIN REGION | BA | SIDE | Z | x | y | z |
| --- | --- | --- | --- | --- | --- | --- |
| RIGHT HEMISPHERE STROKE |  |  |  |  |  |  |
| Lateral Occipital Cortex, superior division | 19 | L | 6.27 | -26 | -64 | 58 |
| Superior Parietal Lobule | 7a | R | 5.95 | 32 | -44 | 50 |
| Occipital Fusiform Gyrus | 37 | R | 5.38 | 24 | -70 | -12 |
| Superior Parietal Lobule | 7a | L | 5.36 | -34 | -42 | 48 |
| Lateral Occipital Cortex, superior division | 19 | R | 5.22 | 32 | -58 | 68 |
| Lingual Gyrus | 19 | R | 5.13 | 16 | -80 | 0 |
| Supramarginal Gyrus, anterior division | 40 | L | 5.13 | -46 | -30 | 40 |
| Temporal Occipital Fusiform Cortex | 20 | L | 5.11 | -40 | -54 | -16 |
| Lateral Occipital Cortex, inferior division | 19 | R | 5.03 | 38 | -74 | 0 |
| Occipital Fusiform Gyrus | 37 | L | 4.93 | -32 | -78 | -16 |
| Temporal Occipital Fusiform Cortex | 20 | R | 4.81 | 30 | -42 | -16 |
| Lateral Occipital Cortex, inferior division | 19 | L | 4.74 | -32 | -82 | -2 |
| Occipital Pole | 17 | L | 4.72 | -16 | -98 | 12 |
| Precentral Gyrus | 4 | R | 4.59 | 42 | -8 | 62 |
| Middle Frontal Gyrus | 46 | L | 4.45 | -34 | -4 | 58 |
| Superior Frontal Gyrus | 6 | L | 3.92 | -24 | -2 | 66 |
| Middle Frontal Gyrus | 46 | R | 3.77 | 26 | 0 | 48 |
| Superior Frontal Gyrus | 6 | R | 3.76 | 28 | 8 | 60 |
| LEFT HEMISPHERE STROKE |  |  |  |  |  |  |
| Temporal Occipital Fusiform Cortex | 20 | R | 5.69 | 28 | -58 | -12 |
| Intracalcarine Cortex | 17 | R | 5.62 | 14 | -82 | 12 |
| Lateral Occipital Cortex, inferior division | 19 | L | 5.58 | -42 | -80 | 6 |
| Superior Parietal Lobule | 7a | R | 5.5 | 34 | -50 | 60 |
| Temporal Occipital Fusiform Cortex | 20 | L | 5.37 | -32 | -48 | -22 |
| Lateral Occipital Cortex, superior division | 19 | L | 5.32 | -20 | -72 | 52 |
| Precentral Gyrus | 4 | R | 4.9 | 36 | -6 | 60 |
| Superior Frontal Gyrus | 6 | L | 4.78 | -24 | -8 | 60 |
| Precentral Gyrus | 4 | L | 4.67 | -24 | -10 | 54 |
| Superior Frontal Gyrus | 6 | R | 4.64 | 26 | -4 | 54 |
| Middle Frontal Gyrus | 46 | L | 3.73 | -32 | -4 | 52 |
| NONDISABLED CONTROLS |  |  |  |  |  |  |
| Occipital Fusiform Gyrus | 37 | L | 5.25 | -32 | -82 | -12 |
| Lateral Occipital Cortex, inferior division | 19 | L | 5.24 | -38 | -78 | -12 |
| Lateral Occipital Cortex, inferior division | 19 | R | 5.22 | 38 | -70 | -4 |
| Temporal Occipital Fusiform Cortex | 20 | L | 5.1 | -40 | -60 | -10 |
| Occipital Pole | 17 | R | 5.05 | 10 | -90 | 14 |
| Superior Parietal Lobule | 7a | R | 4.97 | 30 | -54 | 58 |
| Temporal Occipital Fusiform Cortex | 20 | R | 4.96 | 36 | -44 | -16 |
| Superior Parietal Lobule | 5 | L | 4.95 | -28 | -44 | 50 |
| Supramarginal Gyrus, posterior division | 40 | R | 4.93 | 44 | -36 | 50 |
| Superior Frontal Gyrus | 6 | R | 4.87 | 22 | -6 | 62 |
| Precentral Gyrus | 4 | L | 4.56 | -28 | -8 | 54 |
| Thalamus | 23b | L | 4.39 | -20 | -30 | 2 |
| Thalamus | 23b | R | 4.32 | 18 | -24 | 8 |
| Precentral Gyrus | 4 | R | 4.29 | 28 | -2 | 46 |
| Juxtapositional Lobule Cortex (formerly Supplementary Motor Cortex) | 6 | L | 3.92 | -6 | 4 | 56 |
| Middle Frontal Gyrus | 46 | R | 3.88 | 40 | 0 | 60 |
| Inferior Frontal Gyrus, pars opercularis | 44 | R | 3.76 | 38 | 10 | 24 |
| MNI coordinates, Z>3.1; cluster threshold *p*<0.05 | | | |  |  |  |

**Table S3. Brain activity during right hand action observation versus left hand action observation (RHAO > LHAO).** Regions showing greater activity during right hand action observation than left hand action observation for participants with right hemisphere stroke, participants with left hemisphere stroke, and nondisabled controls

| BRAIN REGION | BA | | SIDE | | Z | | x | | y | | z | |
| --- | --- | --- | --- | --- | --- | --- | --- | --- | --- | --- | --- | --- |
| RIGHT HEMISPHERE STROKE | |  | |  | |  | |  | |  | |  |
| Superior Parietal Lobule | 7a | | L | | 3.78 | | -34 | | -44 | | 62 | |
| Postcentral Gyrus | 2, 3 | | L | | 3.65 | | -42 | | -34 | | 48 | |
| LEFT HEMISPHERE STROKE | |  | |  | |  | |  | |  | |  |
| Precentral Gyrus | 4 | | L | | 4.04 | | -38 | | -6 | | 56 | |
| Superior Parietal Lobule | 7a | | L | | 3.88 | | -36 | | -40 | | 58 | |
| Postcentral Gyrus | 2, 3 | | L | | 3.43 | | -40 | | -36 | | 70 | |
| NONDISABLED CONTROL | |  | |  | |  | |  | |  | |  |
| None above threshold. |  | |  | |  | |  | |  | |  | |
| MNI coordinates, Z>3.1; cluster threshold *p*<0.05 | | | | | |  | |  | |  | |  |

**Table S4. Brain activity during left hand action observation versus right hand action observation (LHAO > RHAO).** Regions showing greater activity during left hand action observation than right hand action observation for participants with right hemisphere stroke, participants with left hemisphere stroke, and nondisabled controls

| BRAIN REGION | BA | SIDE | | Z | | x | | y | | z | |  |
| --- | --- | --- | --- | --- | --- | --- | --- | --- | --- | --- | --- | --- |
| RIGHT HEMISPHERE STROKE | | |  | |  | |  | |  | |  | |
| Intracalcarine Cortex | 17 | R | | 4.43 | | 10 | | -76 | | 4 | |  |
| Lingual Gyrus | 19 | R | | 4.43 | | 4 | | -78 | | 0 | |  |
| Occipital Pole | 19 | R | | 4.22 | | 4 | | -90 | | 32 | |  |
| Lateral Occipital Cortex, superior division | 19 | R | | 3.97 | | 18 | | -86 | | 38 | |  |
| LEFT HEMISPHERE STROKE |  |  | |  | |  | |  | |  | |  |
| Intracalcarine Cortex | 17 | R | | 5.71 | | 10 | | -88 | | 8 | |  |
| Occipital Pole | 19 | R | | 5.7 | | 6 | | -90 | | 2 | |  |
| Lingual Gyrus | 19 | R | | 5.27 | | 10 | | -68 | | 4 | |  |
| Lateral Occipital Cortex, inferior division | 19 | R | | 4.1 | | 48 | | -66 | | 14 | |  |
| NON-DISABLED CONTROL |  |  | |  | |  | |  | |  | |  |
| Occipital Pole | 19 | R | | 5.08 | | 20 | | -88 | | 28 | |  |
| Lingual Gyrus | 19 | R | | 4.95 | | 14 | | -74 | | -4 | |  |
| Intracalcarine Cortex | 17 | R | | 4.77 | | 14 | | -82 | | 6 | |  |
| Lateral Occipital Cortex, inferior division | 19 | R | | 4.32 | | 44 | | -70 | | 2 | |  |
| Superior Parietal Lobule | 7a | R | | 4.26 | | 28 | | -48 | | 62 | |  |
| Postcentral Gyrus | 2,3 | R | | 3.58 | | 34 | | -34 | | 48 | |  |
| MNI coordinates, Z>3.1; cluster threshold *p*<0.05 | | | | | | |  | |  | |  | |

**Table S5. Group Laterality Index Values.** The average of the laterality (LI) index values calculated at different z-values (1.0, 1.5, 2.3) for each region of interest and during right hand action observation (RHAO) and left hand action observation (LHAO). **Top:** Participants with right hemisphere stroke (RHS); **Middle:** participants with left hemisphere stroke (LHS); **Bottom:** non-disabled control participants (NDC).

| Group | *Z* | Inferior frontal gyrus  Pars Opercularis | | Inferior frontal gyrus  Pars Triangularis | | Supramarginal gyrus | | Precentral gyrus | |
| --- | --- | --- | --- | --- | --- | --- | --- | --- | --- |
|  |  | RHAO | LHAO | RHAO | LHAO | RHAO | LHAO | RHAO | LHAO |
| RHS | 1.00 | 0.28 | -0.06 | 0.17 | -0.06 | 0.36 | 0.20 | 0.18 | -0.12 |
|  | 1.50 | 0.36 | -0.12 | 0.09 | -0.18 | 0.37 | 0.21 | 0.24 | -0.15 |
|  | 2.30 | 0.24 | -0.15 | 0.30 | -0.33 | 0.47 | 0.23 | 0.25 | -0.23 |
|  | average | 0.33 | -0.10 | 0.16 | -0.09 | 0.40 | 0.21 | 0.22 | -0.17 |
| LHS | 1.00 | 0.29 | -0.02 | 0.23 | 0.05 | 0.31 | 0.09 | 0.13 | -0.16 |
|  | 1.50 | 0.25 | -0.07 | 0.28 | 0.02 | 0.32 | 0.10 | 0.16 | -0.21 |
|  | 2.30 | 0.19 | -0.07 | 0.19 | 0.03 | 0.32 | 0.02 | 0.16 | -0.32 |
|  | average | 0.28 | -0.05 | 0.24 | 0.01 | 0.32 | 0.07 | 0.15 | -0.23 |
| NDC | 1.00 | -0.12 | -0.07 | -0.18 | -0.04 | -0.07 | 0.08 | -0.08 | -0.08 |
|  | 1.50 | -0.15 | -0.19 | -0.31 | -0.09 | -0.08 | 0.07 | -0.08 | -0.13 |
|  | 2.30 | -0.17 | -0.23 | -0.33 | -0.26 | -0.06 | 0.01 | -0.08 | -0.23 |
|  | average | -0.15 | -0.17 | -0.21 | -0.11 | -0.07 | 0.05 | -0.08 | -0.15 |

**Table S6. Individual Laterality Index Values – Inferior Frontal Gyrus, Pars Opercularis.** The laterality (LI) index values calculated at different z-values (1.0, 1.5, 2.3) for each participant (n=12 per group) during right hand action observation (RHAO) and left hand action observation (LHAO). **Top:** Participants with right hemisphere stroke (RHS); **Middle:** participants with left hemisphere stroke (LHS); **Bottom:** non-disabled control participants (NDC). NaN values indicate both left and right ROIs had 0 voxels survive the given z-threshold.

|  | INFERIOR FRONTAL GYRUS, PARS OPERCULARIS | | | | | | | |
| --- | --- | --- | --- | --- | --- | --- | --- | --- |
|  | RHAO | | | | LHAO | | | |
|  | z=1.0 | z=1.5 | z=2.3 | z-mean | z=1.0 | z=1.5 | z=2.3 | z-mean |
| RHS | | | | | | | | |
| 1 | 0.66 | 0.74 | 0.97 | 0.79 | -0.61 | -0.78 | -1.00 | -0.80 |
| 2 | -0.62 | -0.85 | -1.00 | -0.82 | -0.38 | -0.88 | -1.00 | -0.75 |
| 3 | 0.42 | 0.88 | 1.00 | 0.77 | 0.21 | 0.62 | 0.94 | 0.59 |
| 4 | 0.82 | 0.98 | 1.00 | 0.94 | 0.81 | 0.93 | 1.00 | 0.91 |
| 5 | 0.94 | 1.00 | NaN | 0.97 | 0.82 | 1.00 | NaN | 0.91 |
| 6 | 0.15 | 0.18 | 0.27 | 0.20 | 0.13 | 0.09 | 0.03 | 0.08 |
| 7 | -0.05 | 0.00 | 0.00 | -0.02 | -0.23 | -0.38 | -0.59 | -0.40 |
| 8 | 0.17 | 0.45 | 1.00 | 0.54 | -0.86 | -0.87 | -0.86 | -0.86 |
| 9 | 0.15 | 0.12 | 0.05 | 0.11 | 0.08 | 0.03 | -0.07 | 0.01 |
| 10 | -0.22 | -0.43 | -0.79 | -0.48 | -0.33 | -0.39 | -0.20 | -0.31 |
| 11 | 0.57 | 1.00 | NaN | 0.79 | -0.60 | -1.00 | NaN | -0.80 |
| 12 | 0.32 | 0.19 | -0.12 | 0.13 | 0.20 | 0.16 | 0.21 | 0.19 |
| LHS | | | | | | | | |
| 1 | 0.85 | NaN | NaN | 0.85 | -0.48 | -0.92 | -1.00 | -0.80 |
| 2 | -0.07 | -0.08 | -0.18 | -0.11 | -0.63 | -0.82 | -0.98 | -0.39 |
| 3 | -0.02 | -0.13 | -0.54 | -0.23 | 0.66 | 0.90 | 1.00 | 0.25 |
| 4 | -0.04 | -0.09 | -0.16 | -0.10 | 0.10 | 0.08 | 0.06 | -0.03 |
| 5 | 0.18 | 0.29 | 0.48 | 0.32 | 0.52 | 0.66 | 0.76 | 0.42 |
| 6 | 0.58 | 0.72 | 0.86 | 0.72 | 0.40 | 0.57 | 0.71 | 0.67 |
| 7 | -0.53 | -0.80 | -1.00 | -0.78 | -0.90 | -1.00 | -1.00 | -0.77 |
| 8 | 0.89 | 1.00 | 1.00 | 0.96 | -0.40 | -0.33 | 1.00 | 0.95 |
| 9 | 0.61 | 0.70 | 0.58 | 0.63 | 0.15 | 0.13 | 0.13 | 0.46 |
| 10 | 0.57 | 0.62 | 0.66 | 0.62 | 0.20 | 0.05 | -1.00 | 0.06 |
| 11 | 0.70 | 0.86 | 1.00 | 0.85 | 0.59 | 0.51 | 0.35 | 0.63 |
| 12 | -0.28 | -0.38 | -0.57 | -0.41 | -0.50 | -0.63 | -0.91 | -0.54 |
| ND | | | | | | | | |
| 1 | -0.31 | -0.51 | -0.77 | -0.53 | -0.24 | -0.41 | -0.64 | -0.43 |
| 2 | -0.86 | -0.96 | -1.00 | -0.94 | -0.11 | -0.43 | -0.92 | -0.49 |
| 3 | -0.91 | -1.00 | -1.00 | -0.97 | -0.93 | -1.00 | -1.00 | -0.98 |
| 4 | -0.99 | -1.00 | -1.00 | -1.00 | -0.18 | -1.00 | NaN | -0.59 |
| 5 | 0.03 | 0.06 | 0.09 | 0.06 | 0.05 | 0.05 | -0.02 | 0.02 |
| 6 | 0.29 | 0.30 | 0.18 | 0.25 | 0.33 | 0.32 | 0.30 | 0.32 |
| 7 | -0.01 | -0.06 | 0.04 | -0.01 | -0.20 | -0.11 | 0.06 | -0.08 |
| 8 | 0.60 | 0.65 | 0.74 | 0.66 | 0.35 | 0.42 | 0.42 | 0.39 |
| 9 | 0.66 | 0.68 | 0.72 | 0.69 | 0.44 | 0.53 | 0.51 | 0.49 |
| 10 | 0.14 | 0.17 | 0.18 | 0.16 | 0.26 | 0.28 | 0.12 | 0.22 |
| 11 | -0.14 | -0.21 | -0.30 | -0.22 | -0.05 | -0.13 | -0.34 | -0.17 |
| 12 | 0.05 | 0.08 | 0.13 | 0.09 | -0.59 | -0.75 | -0.96 | -0.77 |

**Table S7. Individual Laterality Index Values – Inferior Frontal Gyrus, Pars Triangularis.** The laterality (LI) index values calculated at different z-values (1.0, 1.5, 2.3) for each participant (n=12 per group) during right hand action observation (RHAO) and left hand action observation (LHAO). **Top:** Participants with right hemisphere stroke (RHS); **Middle:** participants with left hemisphere stroke (LHS); **Bottom:** non-disabled control participants (NDC). NaN values indicate both left and right ROIs had 0 voxels survive the given z-threshold.

|  | INFERIOR FRONTAL GYRUS, PARS TRIANGULARIS | | | | | | | |
| --- | --- | --- | --- | --- | --- | --- | --- | --- |
|  | RHAO | | | | LHAO | | | |
|  | z=1.0 | z=1.5 | z=2.3 | z-mean | z=1.0 | z=1.5 | z=2.3 | z-mean |
| RHS | | | | | | | | |
| 1 | 0.26 | -0.67 | NaN | -0.20 | 0.71 | NaN | NaN | 0.71 |
| 2 | -0.53 | -0.61 | -1.00 | -0.71 | -0.75 | -0.86 | NaN | -0.81 |
| 3 | -0.70 | -1.00 | NaN | -0.85 | -0.74 | -0.82 | NaN | -0.78 |
| 4 | 0.24 | 0.69 | 1.00 | 0.64 | 0.92 | 1.00 | NaN | 0.96 |
| 5 | 0.76 | 0.92 | 1.00 | 0.89 | 0.24 | -0.38 | NaN | -0.07 |
| 6 | 0.19 | 0.22 | 0.22 | 0.21 | 0.25 | 0.25 | 0.24 | 0.25 |
| 7 | 0.25 | -0.13 | 1.00 | 0.37 | -0.58 | -0.80 | -0.97 | -0.78 |
| 8 | 0.39 | 0.63 | 0.25 | 0.42 | -0.96 | -1.00 | -1.00 | -0.99 |
| 9 | 0.20 | 0.23 | 0.18 | 0.20 | 0.03 | -0.02 | -0.13 | -0.04 |
| 10 | -0.69 | -0.87 | -0.99 | -0.85 | -0.92 | -0.98 | -1.00 | -0.97 |
| 11 | 1.00 | 1.00 | NaN | 1.00 | 0.65 | 1.00 | NaN | 0.83 |
| 12 | 0.66 | 0.69 | 1.00 | 0.78 | 0.41 | 0.57 | 0.90 | 0.63 |
| LHS | | | | | | | | |
| 1 | -0.62 | -1.00 | NaN | -0.81 | -0.53 | -0.94 | NaN | -0.73 |
| 2 | -0.21 | -0.06 | -0.80 | -0.36 | -0.67 | -0.83 | -0.95 | -0.82 |
| 3 | -0.78 | -0.89 | -1.00 | -0.89 | -0.83 | -1.00 | NaN | -0.91 |
| 4 | -0.48 | -0.57 | -0.62 | -0.55 | 0.16 | 0.17 | -0.11 | 0.07 |
| 5 | 0.12 | 0.25 | 0.56 | 0.31 | 0.24 | 0.24 | 1.00 | 0.49 |
| 6 | 0.12 | 0.16 | -0.23 | 0.02 | 0.44 | 0.59 | 0.42 | 0.48 |
| 7 | 0.69 | 0.87 | NaN | 0.78 | -1.00 | -1.00 | NaN | -1.00 |
| 8 | 0.92 | 0.98 | 1.00 | 0.97 | 0.31 | 0.45 | -1.00 | -0.08 |
| 9 | 1.00 | 1.00 | 1.00 | 1.00 | 0.81 | 0.82 | NaN | 0.81 |
| 10 | 0.51 | 0.62 | 0.75 | 0.63 | 0.89 | 0.90 | 1.00 | 0.93 |
| 11 | 0.87 | 1.00 | NaN | 0.93 | 0.87 | 1.00 | NaN | 0.93 |
| 12 | 0.58 | 0.96 | 1.00 | 0.85 | -0.10 | -0.12 | -0.12 | -0.12 |
| ND | | | | | | | | |
| 1 | -0.73 | -0.81 | -1.00 | -0.85 | -0.39 | -0.75 | -0.90 | -0.68 |
| 2 | -0.71 | -0.78 | NaN | -0.74 | -0.05 | -0.24 | -0.71 | -0.33 |
| 3 | -0.92 | -1.00 | -1.00 | -0.97 | -0.15 | 0.10 | -0.33 | -0.13 |
| 4 | -0.96 | -0.98 | -1.00 | -0.98 | 0.37 | 0.36 | NaN | 0.37 |
| 5 | -0.22 | -0.40 | -0.92 | -0.51 | -0.14 | -0.15 | -0.23 | -0.17 |
| 6 | 0.43 | 0.45 | 0.38 | 0.42 | 0.46 | 0.48 | 0.43 | 0.46 |
| 7 | 0.13 | 0.19 | 0.51 | 0.28 | 0.06 | 0.32 | 0.84 | 0.41 |
| 8 | -0.07 | -0.12 | -0.17 | -0.12 | -0.03 | 0.06 | 0.09 | 0.04 |
| 9 | 1.00 | NaN | NaN | 1.00 | 0.07 | -0.26 | -0.50 | -0.23 |
| 10 | -0.23 | -0.21 | -0.04 | -0.16 | 0.04 | 0.16 | 0.42 | 0.20 |
| 11 | 0.25 | 0.42 | 0.43 | 0.37 | -0.24 | -0.38 | -1.00 | -0.54 |
| 12 | -0.13 | -0.22 | -0.48 | -0.27 | -0.49 | -0.72 | -0.94 | -0.72 |

**Table S8. Individual Laterality Index Values – Supramarginal Gyrus.** The laterality (LI) index values calculated at different z-values (1.0, 1.5, 2.3) for each participant (n=12 per group) during right hand action observation (RHAO) and left hand action observation (LHAO). **Top:** Participants with right hemisphere stroke (RHS); **Middle:** participants with left hemisphere stroke (LHS); **Bottom:** non-disabled control participants (NDC). NaN values indicate both left and right ROIs had 0 voxels survive the given z-threshold.

|  | SUPRAMARGINAL GYRUS | | | | | | | |
| --- | --- | --- | --- | --- | --- | --- | --- | --- |
|  | RHAO | | | | LHAO | | | |
|  | z=1.0 | z=1.5 | z=2.3 | z-mean | z=1.0 | z=1.5 | z=2.3 | z-mean |
| RHS | | | | | | | | |
| 1 | 0.04 | 0.06 | 0.08 | 0.06 | 0.01 | 0.00 | 0.00 | 0.00 |
| 2 | -0.95 | -1.00 | -1.00 | -0.98 | -0.06 | -0.24 | -0.68 | -0.32 |
| 3 | -0.24 | -0.30 | -0.34 | -0.29 | -0.03 | -0.13 | -0.38 | -0.18 |
| 4 | -0.91 | -0.99 | -1.00 | -0.97 | 0.58 | 0.71 | 1.00 | 0.76 |
| 5 | -0.09 | -0.11 | -0.10 | -0.10 | -0.08 | -0.16 | -0.28 | -0.17 |
| 6 | 0.41 | 0.50 | 0.55 | 0.49 | 0.29 | 0.31 | 0.32 | 0.30 |
| 7 | -0.08 | -0.20 | -0.32 | -0.20 | -0.31 | -0.34 | -0.36 | -0.34 |
| 8 | 0.14 | 0.17 | 0.22 | 0.18 | 0.15 | 0.20 | 0.26 | 0.20 |
| 9 | 0.59 | 0.63 | 0.79 | 0.67 | 0.25 | 0.30 | 0.36 | 0.30 |
| 10 | 0.06 | 0.08 | 0.19 | 0.11 | 0.09 | 0.01 | -0.17 | -0.02 |
| 11 | 0.20 | 0.23 | 0.29 | 0.24 | 0.24 | 0.28 | 0.34 | 0.28 |
| 12 | -0.05 | -0.08 | -0.14 | -0.09 | -0.14 | -0.21 | -0.33 | -0.23 |
| LHS | | | | | | | | |
| 1 | 0.97 | 1.00 | 1.00 | 0.99 | -0.46 | -0.23 | -1.00 | -0.56 |
| 2 | 0.24 | 0.31 | 0.42 | 0.32 | 0.01 | -0.01 | -0.16 | -0.05 |
| 3 | 0.93 | 1.00 | 1.00 | 0.98 | 0.95 | 0.98 | 1.00 | 0.98 |
| 4 | -0.08 | -0.12 | -0.20 | -0.13 | -0.01 | -0.03 | -0.07 | -0.04 |
| 5 | 0.20 | 0.22 | 0.27 | 0.23 | 0.43 | 0.48 | 0.55 | 0.49 |
| 6 | 0.13 | 0.18 | 0.41 | 0.24 | 0.07 | 0.10 | 0.24 | 0.14 |
| 7 | 0.00 | -0.05 | -0.15 | -0.07 | -0.12 | -0.15 | -0.20 | -0.16 |
| 8 | 0.73 | 0.80 | 0.90 | 0.81 | 0.53 | 0.60 | 0.70 | 0.61 |
| 9 | 0.30 | 0.08 | -0.69 | -0.10 | -0.29 | -0.51 | -0.93 | -0.58 |
| 10 | 0.08 | 0.12 | 0.21 | 0.14 | 0.09 | 0.12 | 0.11 | 0.11 |
| 11 | 0.14 | 0.26 | 0.62 | 0.34 | 0.21 | 0.33 | 0.55 | 0.36 |
| 12 | 0.12 | 0.09 | 0.01 | 0.07 | -0.36 | -0.43 | -0.51 | -0.43 |
| ND | | | | | | | | |
| 1 | 0.04 | 0.06 | 0.08 | 0.06 | 0.01 | 0.00 | 0.00 | 0.00 |
| 2 | -0.95 | -1.00 | -1.00 | -0.98 | -0.06 | -0.24 | -0.68 | -0.32 |
| 3 | -0.24 | -0.30 | -0.34 | -0.29 | -0.03 | -0.13 | -0.38 | -0.18 |
| 4 | -0.91 | -0.99 | -1.00 | -0.97 | 0.58 | 0.71 | 1.00 | 0.76 |
| 5 | -0.09 | -0.11 | -0.10 | -0.10 | -0.08 | -0.16 | -0.28 | -0.17 |
| 6 | 0.41 | 0.50 | 0.55 | 0.49 | 0.29 | 0.31 | 0.32 | 0.30 |
| 7 | -0.08 | -0.20 | -0.32 | -0.20 | -0.31 | -0.34 | -0.36 | -0.34 |
| 8 | 0.14 | 0.17 | 0.22 | 0.18 | 0.15 | 0.20 | 0.26 | 0.20 |
| 9 | 0.59 | 0.63 | 0.79 | 0.67 | 0.25 | 0.30 | 0.36 | 0.30 |
| 10 | 0.06 | 0.08 | 0.19 | 0.11 | 0.09 | 0.01 | -0.17 | -0.02 |
| 11 | 0.20 | 0.23 | 0.29 | 0.24 | 0.24 | 0.28 | 0.34 | 0.28 |
| 12 | -0.05 | -0.08 | -0.14 | -0.09 | -0.14 | -0.21 | -0.33 | -0.23 |

**Table S9. Individual Laterality Index Values – Precentral Gyrus.** The laterality (LI) index values calculated at different z-values (1.0, 1.5, 2.3) for each participant (n=12 per group) during right hand action observation (RHAO) and left hand action observation (LHAO). **Top:** Participants with right hemisphere stroke (RHS); **Middle:** participants with left hemisphere stroke (LHS); **Bottom:** non-disabled control participants (NDC). NaN values indicate both left and right ROIs had 0 voxels survive the given z-threshold.

|  | PRECENTRAL GYRUS | | | | | | | |
| --- | --- | --- | --- | --- | --- | --- | --- | --- |
|  | RHAO | | | | LHAO | | | |
|  | z=1.0 | z=1.5 | z=2.3 | z-mean | z=1.0 | z=1.5 | z=2.3 | z-mean |
| RHS | | | | | | | | |
| 1 | 0.48 | 0.55 | 0.67 | 0.45 | -0.35 | -0.55 | -0.89 | -0.60 |
| 2 | -0.05 | -0.03 | 0.02 | -0.05 | -0.23 | -0.27 | -0.28 | -0.26 |
| 3 | 0.18 | 0.25 | 0.34 | 0.20 | -0.09 | -0.11 | -0.20 | -0.13 |
| 4 | 0.39 | 0.50 | 0.64 | 0.44 | 0.01 | -0.01 | -0.10 | -0.03 |
| 5 | 0.47 | 0.58 | 0.66 | 0.53 | 0.45 | 0.45 | 0.25 | 0.38 |
| 6 | 0.04 | 0.08 | 0.12 | 0.26 | -0.11 | -0.14 | -0.18 | -0.14 |
| 7 | 0.13 | 0.20 | 0.25 | 0.12 | -0.12 | -0.13 | -0.15 | -0.13 |
| 8 | 0.26 | 0.44 | 0.68 | 0.35 | -0.06 | -0.02 | 0.06 | -0.01 |
| 9 | 0.08 | 0.08 | 0.05 | 0.26 | -0.06 | -0.07 | -0.10 | -0.08 |
| 10 | -0.26 | -0.34 | -0.46 | 0.02 | -0.24 | -0.27 | -0.36 | -0.29 |
| 11 | 0.09 | 0.01 | -0.57 | 0.07 | -0.79 | -0.92 | -0.98 | -0.90 |
| 12 | 0.41 | 0.52 | 0.64 | 0.40 | 0.19 | 0.20 | 0.17 | 0.19 |
| LHS | | | | | | | | |
| 1 | 0.61 | 0.78 | 0.92 | 0.77 | -0.51 | -0.67 | -1.00 | -0.73 |
| 2 | -0.13 | -0.14 | -0.14 | -0.13 | -0.44 | -0.55 | -0.75 | -0.58 |
| 3 | 0.15 | 0.15 | -0.09 | 0.07 | 0.32 | 0.39 | 0.44 | 0.38 |
| 4 | -0.02 | 0.01 | 0.04 | 0.01 | -0.11 | -0.13 | -0.14 | -0.13 |
| 5 | 0.02 | 0.06 | 0.12 | 0.07 | 0.13 | 0.15 | 0.12 | 0.14 |
| 6 | 0.06 | 0.07 | 0.02 | 0.05 | -0.10 | -0.10 | -0.21 | -0.14 |
| 7 | -0.15 | -0.16 | -0.14 | -0.15 | -0.35 | -0.45 | -0.71 | -0.50 |
| 8 | 0.40 | 0.61 | 0.82 | 0.61 | -0.08 | -0.06 | -0.04 | -0.06 |
| 9 | 0.23 | 0.27 | 0.17 | 0.22 | -0.27 | -0.49 | -0.73 | -0.50 |
| 10 | 0.07 | 0.07 | 0.01 | 0.05 | -0.23 | -0.26 | -0.29 | -0.26 |
| 11 | 0.29 | 0.30 | 0.32 | 0.30 | 0.14 | 0.09 | 0.03 | 0.08 |
| 12 | 0.00 | -0.03 | -0.08 | -0.04 | -0.40 | -0.45 | -0.51 | -0.45 |
| ND | | | | | | | | |
| 1 | 0.07 | 0.08 | 0.09 | 0.08 | -0.09 | -0.11 | -0.11 | -0.11 |
| 2 | -0.66 | -0.75 | -0.78 | -0.73 | -0.18 | -0.28 | -0.48 | -0.32 |
| 3 | -0.24 | -0.31 | -0.45 | -0.33 | -0.27 | -0.38 | -0.56 | -0.41 |
| 4 | -0.84 | -0.90 | -0.90 | -0.88 | 0.07 | -0.16 | -0.56 | -0.22 |
| 5 | 0.01 | 0.03 | 0.05 | 0.03 | -0.12 | -0.13 | -0.17 | -0.14 |
| 6 | 0.26 | 0.25 | 0.19 | 0.23 | 0.15 | 0.07 | -0.07 | 0.05 |
| 7 | -0.12 | -0.08 | 0.02 | -0.06 | -0.28 | -0.27 | -0.25 | -0.27 |
| 8 | 0.20 | 0.21 | 0.23 | 0.22 | 0.03 | 0.03 | 0.02 | 0.03 |
| 9 | 0.37 | 0.42 | 0.45 | 0.41 | 0.04 | 0.05 | 0.00 | 0.03 |
| 10 | 0.00 | 0.02 | 0.05 | 0.02 | -0.17 | -0.23 | -0.35 | -0.25 |
| 11 | 0.02 | 0.02 | 0.04 | 0.03 | -0.10 | -0.12 | -0.16 | -0.13 |
| 12 | -0.01 | 0.01 | 0.04 | 0.01 | -0.06 | -0.05 | -0.01 | -0.04 |
